# Targeting mechanosensitive EphA2 phase separation to alleviate arterial stiffening

**DOI:** 10.1016/j.bioactmat.2026.01.020

**Published:** 2026-01-24

**Authors:** Jia-Yu Liu, Geng Shen, Yi-Chen Lin, Jing Chen, Qin-Ye Chen, Mo-Jun Lin

**Affiliations:** a Department of Physiology and Pathophysiology, School of Basic Medical Sciences, Key Laboratory of Fujian Province Universities on Ion Channel and Signal Transduction in Cardiovascular Diseases, Fujian Medical University, Fuzhou, 350122, China; b Department of Cardiology, Beijing Anzhen Hospital, Capital Medical University, Beijing, 100029, China; c Department of Vascular Surgery, The First Affiliated Hospital, Fujian Medical University, Fuzhou, 350005, China; d Department of Vascular Surgery, National Regional Medical Center, Binhai Campus of the First Affiliated Hospital, Fujian Medical University, Fuzhou, 350212, China

**Keywords:** Matrix stiffness, Phase separation, Vascular smooth muscle, EphA2, Arterial stiffening

## Abstract

Arterial stiffening, a major cardiovascular risk factor, is driven by aberrant mechanotransduction in vascular smooth muscle cells (VSMCs), yet the critical mechanoreceptors and underlying mechanisms remain elusive. Here, we identified Ephrin receptor A2 (EphA2) as a significantly upregulated mechanosensitive receptor in stiffened arteries from a 5/6 nephrectomy mouse model. Genetic deletion of Epha2 in VSMCs markedly attenuated arterial stiffening. Utilizing polyacrylamide gels of varying stiffness and in situ stiffening bioclick hydrogels, we demonstrated that matrix stiffening directly induces EphA2 phase separation, forming a biomolecular condensate that serves as a signaling hub to recruit and activate ERK1/2. This leads to phosphorylation of the transcription factor CREB and subsequent upregulation of the pro-remodeling nuclear receptor NR4A3. To translate this discovery, we designed a retro-reversed peptide targeting the intrinsically disordered regions (IDRs) of EphA2, which effectively disrupted phase separation and mitigated VSMCs dysfunction in vitro. Crucially, in vivo delivery of this peptide via VAPG-modified nanoparticles significantly alleviated arterial calcification and stiffening in mice. Our study establishes EphA2 phase separation as a pivotal mechanism in vascular mechanotransduction and unveils a novel EphA2-ERK1/2-NR4A3 signaling axis, thereby presenting a promising therapeutic strategy for combating arterial stiffening by targeting pathological biomolecular condensates.

## Highlights

• EphA2 forms mechanosensitive biomolecular condensates that drive arterial stiffening via ERK1/2-CREB-NR4A3 signaling.
• A retro‑reversed peptide targeting EphA2’s disordered region inhibits its phase separation and downstream signaling.
• Nanoparticle-delivered peptide disrupts EphA2 phase separation in vivo, alleviating vascular stiffening and calcification.

## Graphical abstract

**Figure:**
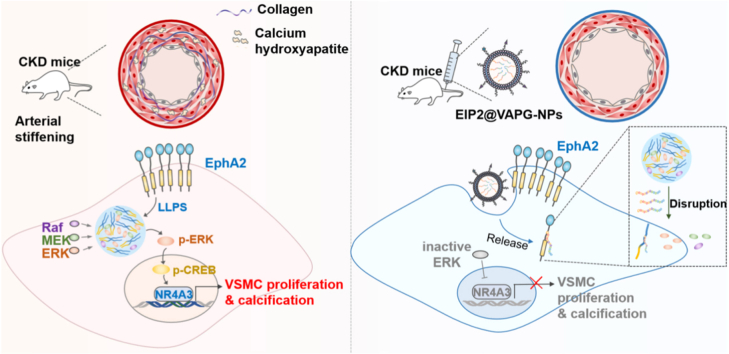

## 1 Introduction

Arterial stiffening occurs in many pathological conditions, including aging, atherosclerosis, hypertension, diabetes and chronic kidney disease (CKD) [1,2]. Increased arterial stiffness is a dominant risk factor for cardiovascular mortality. The stiffness of healthy blood vessels is generally 2–5 kPa, which can dramatically increase in many pathological conditions [3,4]. The stiffened matrix promotes the transformation of vascular smooth muscle cells (VSMCs) from a contractile to a synthetic phenotype, which is characterized by increased proliferation, migration, and synthetic capabilities [5,6]. The mechanoreceptors located on the cell membrane are the first responders to the extracellular mechanical microenvironment [7,8]. Therefore, exploring the molecular mechanisms underlying the development of arterial stiffening, especially identifying mechanosensitive receptors in vascular cells, is crucial for identifying new targets to combat arterial stiffening.

The mechanoreceptors in vascular cells primarily consist of receptor tyrosine kinases (RTKs), integrins, ion channels, and G protein-coupled receptors (GPCRs) [8]. Emerging evidence has shown that RTKs play important roles in mechanotransduction [[9], [10], [11], [12]]. Recently, discoidin domain receptor 1 (DDR1) has been identified as a novel mechanosensor that regulates Yes-associated protein (YAP) activation in endothelial and smooth muscle cells [13,14]. However, the functional roles of other RTKs in cardiovascular pathophysiology remain largely unexplored.

The erythropoietin-producing hepatoma (Eph) receptor family is the largest family of RTKs and is a key regulator of cell growth, differentiation and motility [15]. Emerging evidence indicates that the interaction between the Eph receptor and its ligand plays an important role in cardiovascular physiology and pathology, participating in the regulation of cardiovascular development, angiogenesis, atherosclerosis and cardiac fibrosis [16]. EphA2 is an important subtype of the Eph receptor family that has been widely studied. Several studies have shown that EphA2 can regulate the inflammatory response in atherosclerosis [17,18]. Besides, in breast cancer, EphA2 has been reported to regulate epithelial-mesenchymal transition and metastasis in response to increasing stiffness [19]. However, whether matrix stiffness can regulate EphA2 activation in VSMCs remains unknown, and its downstream signaling pathways need to be elucidated.

Liquid-liquid phase separation (LLPS) is a crucial and ubiquitous phenomenon in many fundamental cell processes, including the formation of membraneless condensates that can improve signaling speed and specificity [20]. A previous study revealed that DDR1 can undergo force-induced LLPS and cocondense with downstream proteins to regulate cellular functions [13,14]. In addition to DDR1, Eph family receptors can also form supramolecular clusters [15]. Intriguingly, when the concentration of Eph receptors on the cell membrane is sufficiently high, they can self-assemble into higher-order oligomers independently of ligand binding [15]. These findings suggest that the increase in Eph receptor expression may facilitate receptor-mediated signaling through oligomerization, bypassing the need for ephrin ligation. Since LLPS is influenced by factors such as protein concentration and posttranslational modifications, Eph receptors might also undergo LLPS under specific conditions [21]. Notably, a recent study revealed that EphA2 is abnormally highly expressed in colorectal cancer tissues and can undergo LLPS to promote tumor progression [22]. Given the upregulation of EphA2 following the transition of VSMCs from a contractile to a synthetic phenotype [18], we hypothesized that this increased expression might similarly drive EphA2 phase separation, potentially regulating VSMC behavior.

Here, we describe an LLPS-dependent mechanism by which EphA2 regulates ERK activation and NR4A3 expression in response to mechanical stimuli. Matrix stiffness promotes EphA2 expression, clustering, and droplet formation, which is independent of its ligand-binding domain. Proteomic identification revealed that EphA2 can recruit ERK1/2 into liquid droplets and create a signaling hub to phosphorylate them. The activation of ERK1/2 can phosphorylate the transcription factor cAMP-response element binding protein (CREB) [23] and subsequently promote the expression of NR4A3 [24], a key nuclear receptor in vascular biology [25]. Disrupting EphA2 phase separation with interfering peptides significantly inhibited the progression of arterial stiffening. Our study reveals an important role of the EphA2-ERK-NR4A3 axis in the mechanotransduction of VSMCs and suggests that therapeutically targeting EphA2 LLPS can be used as an anti-arterial stiffening strategy.

## 2 Results

### 2.1 EphA2 is a critical contributor to arterial stiffening

**Fig. 1:**
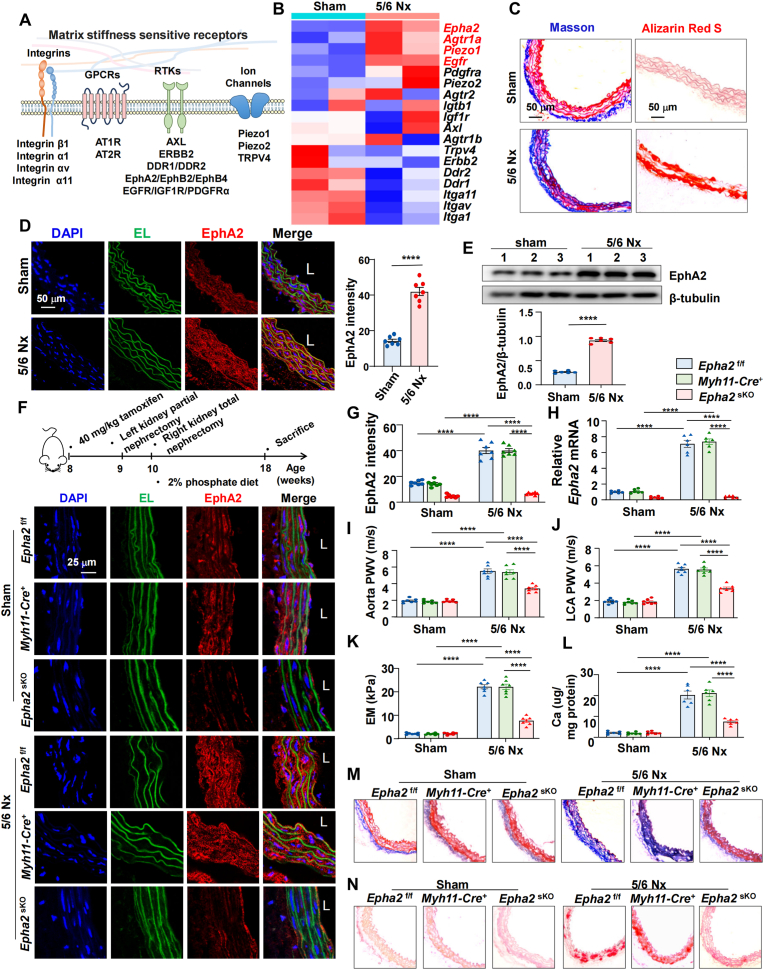
EphA2 is a critical contributor to arterial stiffening. **(A)** Diagram illustrating potential mechanoreceptors for ECM stiffness, including GPCRs, integrins, ion channels and RTKs. **(B)** Heatmap of differentially expressed mRNAs of potential mechanoreceptors in the aortas of sham and 5/6 nephrectomy (Nx) mice. Blue indicates low expression, and red indicates high expression. **(C)** Masson and Alizarin red S staining in the indicated aortas. **(D)** Immunofluorescence of EphA2 in the indicated aortas and quantification of EphA2 intensity. L: lumen. n = 7 mice. **(E)** Western blotting and quantification of EphA2 expression in the aortas of sham and 5/6 Nx mice, n = 6 mice. **(F)** Up: Schematic diagram of the 5/6 nephrectomy (Nx)-induced arterial stiffening model. Down: Immunofluorescence of EphA2 in the aortas of the indicated groups. **(G)** Quantification of EphA2 intensity in the tunica media of aortas from the indicated groups. n = 7 mice. **(H)** Quantitative RT-PCR analysis of Epha2 expression in the tunica media of the indicated mice. n = 7 mice. **(I** and **J)** Pulse wave velocity (PWV) of the aorta and carotid artery in the indicated mice. n = 7 mice. **(K)** The elastic modulus of thoracic aortas from the indicated mice was measured by nanoindentation. n = 7 mice. **(L)** Quantification of calcification deposition in the aortas of the indicated mice, normalized to μg Ca/mg protein. n = 6 mice. **(M** and **N)** Masson and Alizarin red S staining of the thoracic aortas of the indicated mice. The data are expressed as the means ± SEMs and were analyzed by unpaired *t*-test (D and E) or two-way ANOVA followed by Tukey's multiple comparison test (G-L). ∗∗∗∗P < 0.0001.

To identify potential mechanoreceptors involved in arterial stiffening (Fig. 1A) [8], we analyzed publicly available RNA-sequencing data from aortic tissues of 5/6 nephrectomy (Nx) and sham-operated mice, sourced from the GEO database (accession number GSE159833) [26]. A gene expression heatmap highlighted EphA2 as the most markedly upregulated mechanoreceptor in stiffened arteries compared to sham controls (Fig. 1B). To further validate the upregulation of EphA2 during arterial stiffening, we performed 5/6 nephrectomy or sham surgery on C57BL/6J wild-type mice to model chronic kidney disease and subsequent arterial stiffening (Fig. S1A). At eight weeks post-surgery, pulse wave velocity (PWV) measurements in the aorta and carotid artery were significantly elevated in Nx mice (Fig. S1B and S1C), confirming the development of vascular stiffness. Consistent with these findings, nanoindentation revealed a pronounced increase in aortic stiffness in the 5/6 Nx group compared to sham controls (20.1 ± 3.5 kPa vs. 2.0 ± 0.5 kPa) (Fig. S1D). Masson and Alizarin red S staining revealed increased collagen and calcium deposition in the vessel walls of 5/6 Nx mice, respectively (Fig. 1C). Importantly, immunofluorescence staining and Western blot analysis showed a substantial increase in EphA2 expression in Nx arteries (Fig. 1D and E).

To investigate the specific contribution of VSMC-expressed EphA2 to arterial stiffening, we generated VSMC-specific *Epha2*^sKO^ (*Epha2*^flox/flox^, *Myh11-CreERT2*^+^) mice (Fig. S2). We used dual littermate controls to isolate genetic background effects, with *Epha2*^f/f^; *Myh11-CreERT2*^-^ mice controlling for the floxed allele, and *Epha2*^+/+^; *Myh11-CreERT2*^+^ mice controlling for Cre activity. The knockout efficiency was validated by immunofluorescence and quantitative RT-PCR (Fig. 1F–H). The Nx procedure induced a comparable mild reduction in body weight (Fig. S3A), elevated systolic and diastolic blood pressure and increased serum markers of renal dysfunction (BUN and creatinine) (Fig. S3B–E). Importantly, despite the overall hypertensive state, blood pressure was significantly lower in Nx-*Epha2*^sKO^ mice compared to both controls (Fig. S3B–C). Crucially, VSMC-specific deletion of *Epha2* conferred significant protection against arterial stiffening. Pulse wave velocity (PWV) in the aorta and carotid artery was markedly reduced in Nx-*Epha2*^sKO^ mice compared to Nx-controls (Fig. 1I and J). Nanoindentation showed a lower aortic elastic modulus in the *Epha2*^sKO^ mice (Fig. 1K). Calcium colorimetric assays, together with Masson and Alizarin red S staining, demonstrated significantly reduced vascular calcium and collagen deposition in Nx-*Epha2*^sKO^ mice (Fig. 1L–N).

Taken together, these data demonstrate that EphA2 plays a critical role in the progression of arterial stiffening and calcification.

### 2.2 Matrix stiffness promotes EphA2 expression and droplets formation

**Fig. 2:**
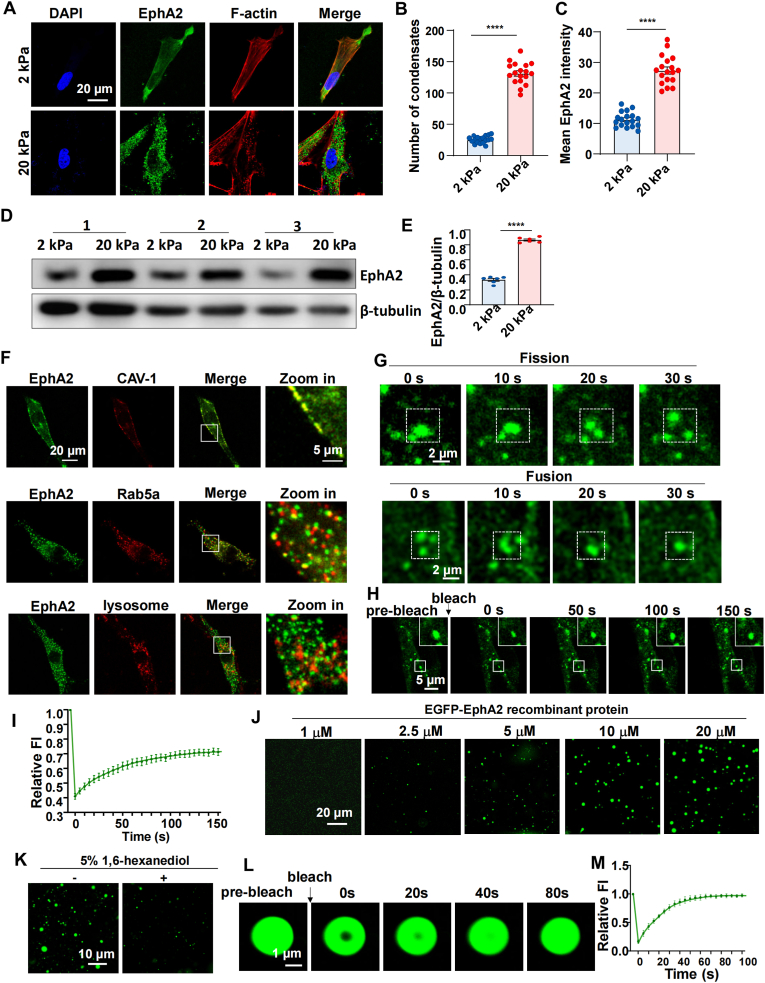
Matrix stiffness promotes EphA2 expression and droplet formation. **(A)** Immunofluorescence of EphA2 and F-actin in HASMCs grown on 2 kPa or 20 kPa gels, respectively. **(B** and **C)** Quantification of the number of EphA2 condensates and EphA2 intensity in **(A)**. The data were analyzed by the Mann-Whitney test. **(D)** Western blotting was used to detect EphA2 expression in HASMCs grown on 2 kPa or 20 kPa gels. **(E)** Quantification of EphA2 expression (relative to β-tubulin) in **(D)**. n = 6 biological replicates. The data were analyzed by unpaired *t*-test. **(F)** Upper: immunofluorescence staining of EphA2 and Lipid raft protein caveolin-1 in HASMCs seeded on 2 kPa kPa gels. Middle: immunofluorescence staining of EphA2 and early endosome marker Rab5A in HASMCs seeded on 20 kPa gels. Lower: live cell imaging of EGFP-EphA2 and LysoTracker Red in HASMCs seeded on 20 kPa gels. **(G)** Live-cell imaging of HASMCs expressing EGFP-EphA2. HASMCs were seeded on 20 kPa gels. EphA2 condenses fusion and fission events, as highlighted in box. **(H)** Images of photobleaching analysis of EGFP-EphA2 condensates in HASMCs. HASMCs were seeded on 20 kPa gels. **(I)** Quantification of fluorescence intensity (FI) recovery in the bleached region of EGFP-EphA2 condensates. n = 9 biological replicates. **(J)** Cell-free phase separation assay showing the droplet formation of EGFP-EphA2 at different concentrations**. (K)** Representative fluorescence microscopy images of droplets formed by 10 μM EGFP-EphA2 before and after treatment with 5 % 1,6-hexanediol. **(L)** FRAP analysis of EGFP-EphA2 (10 μM). **(M)** Quantification of the fluorescence intensity of the bleached area. n = 9 biological replicates. The data are expressed as the means ± SEMs. ∗∗∗∗P < 0.0001.

To examine how matrix stiffness influences EphA2 expression in VSMCs, we cultured human aortic smooth muscle cells (HASMCs) on polyacrylamide (PA) hydrogels with elastic moduli of 2 kPa and 20 kPa, mimicking the mechanical environments of healthy and diseased vessels, respectively (Fig. S1D). Immunofluorescence analysis showed that on soft substrates, EphA2 exhibited a diffuse localization pattern within the cell membrane and cytoplasm. In contrast, on stiff substrates, EphA2 organized into numerous cytoplasmic condensates (Fig. 2A and B). The quantification of fluorescence intensity demonstrated a significant upregulation of EphA2 in cells grown on stiff compared to soft substrates (Fig. 2C), which was confirmed by Western blot analysis (Fig. 2D and E). Further subcellular localization studies revealed that EphA2 was predominantly membrane-associated on soft gels. However, under stiff conditions, EphA2 formed condensates that partially co-localized with the early endosome marker Rab5a, but showed minimal overlap with lysosomal markers (Fig. 2F). This suggests that upon stiffness-induced internalization, EphA2 avoids lysosomal degradation and may instead accumulate as membraneless organelles. Inspired by recent reports identifying EphA2 as a phase separation protein involved in ferroptosis and immune infiltration in colorectal cancer [27], we hypothesized that a similar LLPS mechanism might occur in VSMCs.

Live-cell imaging of HASMCs expressing EGFP-EphA2 confirmed the dynamic behavior of these condensates, showing fusion and fission events on stiff substrates (Fig. 2G). Moreover, fluorescence recovery after photobleaching (FRAP) assays indicated rapid recovery of EphA2 condensates (Fig. 2H and I), supporting their liquid-like properties. To assess whether the mechanosensitivity of EphA2 depends on its ligand binding domain (LBD), we constructed plasmids expressing EGFP-EphA2 or EGFP-EphA2-ΔLBD (Fig. S4A), and utilized in situ stiffening bioclick hydrogels [28] to investigate the response of EphA2 to matrix stiffening. Time-lapse imaging showed that deletion of the LBD did not impair EphA2's ability to form condensates in response to substrate stiffening (Fig. S4B), suggesting that stiffness-induced EphA2 phase separation is independent of ligand binding.

To determine whether EphA2 itself undergoes LLPS in a cell-free system, we purified recombinant EGFP-EphA2 protein and performed in vitro phase separation assays. As expected, EGFP-EphA2 formed liquid-like droplets in a concentration-dependent manner (Fig. 2J). The addition of 1,6-hexanediol, an aliphatic alcohol that disrupts weak hydrophobic interactions, significantly reduced the number of EphA2 droplets (Fig. 2K). Furthermore, FRAP assay revealed that these droplets quickly recovered after fluorescence bleaching (Fig. 2L and M), indicating dynamic liquid characteristics.

Together, these findings demonstrate that increased matrix stiffness not only upregulates EphA2 expression but also drives its LLPS.

### 2.3 Stiffness promoted the interaction and co-condensation of EphA2 and ERK1/2

**Fig. 3:**
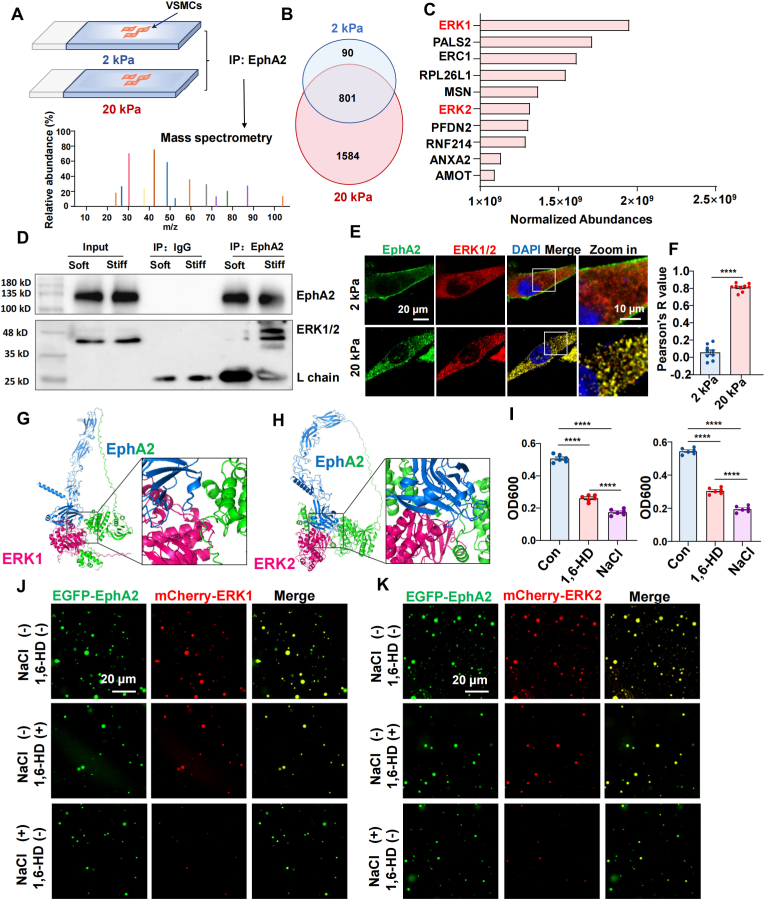
Stiffness promoted the interaction and co-condensation of EphA2 and ERK1/2. **(A)** Schematic diagram of the identification of EphA2-interacting proteins via coimmunoprecipitation (co-IP) and mass spectrometry. HASMCs were seeded on 2 kPa or 20 kPa gels. **(B)** Venn diagram displaying EphA2-interacting proteins identified from the 2 kPa or 20 kPa group. **(C)** The top 10 proteins with the highest abundance uniquely detected in the stiff group were showed. **(D)** co-IP using an anti-EphA2 antibody and light chain-specific secondary antibody to detect the interaction of EphA2 and ERK1/2. **(E)** Representative images of EphA2 and ERK1/2 immunofluorescence in HASMCs seeded on 2 kPa or 20 kPa gels, respectively. **(F)** Colocalization analysis of EphA2 and ERK1/2 in HASMCs seeded on 2 kPa or 20 kPa gels. Pearson's R value (above the threshold) was calculated via ImageJ Fiji software. The data were analyzed by the Mann-Whitney test. **(G** and **H)** Structural models of the EphA2-ERK1/2 complex predicted by AlphaFold3. The extracellular domain of EphA2 is shown in blue, and the transmembrane and intracellular domains are shown in green. ERK1/2 (hot pink) are predicted to interact with multiple sites on both extracellular and intracellular domains of EphA2. **(I)** Turbidity of EGFP-EphA2 (10 μM) and mCherry-ERK1/2 (10 μM) in the presence of 1,6-HD or 150 mM NaCl. n = 6 biological replicates. The data were analyzed by one-way ANOVA followed by Tukey's multiple comparison test. The data are expressed as the means ± SEMs. **(J** and **K)** Representative fluorescence microscopy images of droplets formed by EGFP-EphA2 (10 μM) with mCherry-ERK1/2 (10 μM) in the presence of 1,6-HD or 150 mM NaCl. ∗∗∗∗P < 0.0001.

To identify the signaling pathways downstream of stiffness-induced EphA2 activation, we performed co-immunoprecipitation (co-IP) coupled with mass spectrometry to profile endogenous EphA2-interacting proteins in HASMCs cultured on 2 kPa and 20 kPa substrates (Fig. 3A). Comparative analysis identified 891 proteins in the 2 kPa group and 2385 proteins in the 20 kPa group, with 1584 proteins uniquely detected under stiff conditions (Fig. 3B). Gene Ontology (GO) enrichment analysis revealed that these stiffness-specific interactors were primarily associated with cellular organization, protein metabolism, and cell cycle regulation (Fig. S5). Among these, extracellular signal-regulated kinase 1/2 (ERK1/2) emerged as a high-abundance candidate of interest (Fig. 3C). Subsequent co-IP validation in HASMCs confirmed that substrate stiffness enhances the physical interaction between EphA2 and ERK1/2 (Fig. 3D), a finding supported by increased EphA2-ERK1/2 colocalization observed via immunofluorescence staining (Fig. 3E and F).

To further characterize this interaction, we employed AlphaFold3 for molecular docking, which predicted multiple contact sites between EphA2 and ERK1/2 (Fig. 3G). We next asked whether EphA2 could recruit ERK1/2 into condensates in cell-free system. While recombinant mCherry-ERK1 or mCherry-ERK2 alone did not form condensates (Fig. S6A and S6B), they underwent co-condensation with EphA2 (Fig. 3H–J). To probe the biophysical forces governing these interactions, we introduced NaCl to disrupt electrostatic interactions and 1,6-hexanediol (1,6-HD) to interfere with hydrophobic associations. Treatment with 1,6-HD reduced turbidity, droplet size, and number but did not prevent EphA2-ERK1/2 cocondensation. In contrast, NaCl significantly diminished mixture turbidity, disrupted droplet formation, and abolished cocondensation (Fig. 3H–J), suggesting that EphA2 phase separation is stabilized by both electrostatic and hydrophobic forces, while EphA2-ERK1/2 binding is primarily electrostatically driven.

Collectively, these results demonstrate that matrix stiffness promotes EphA2 phase separation, which in turn recruits ERK1/2 via electrostatic interactions to drive their co-condensation.

### 2.4 EphA2 condensation facilitate ERK1/2 recruitment and activation by creating a signaling hub

**Fig. 4:**
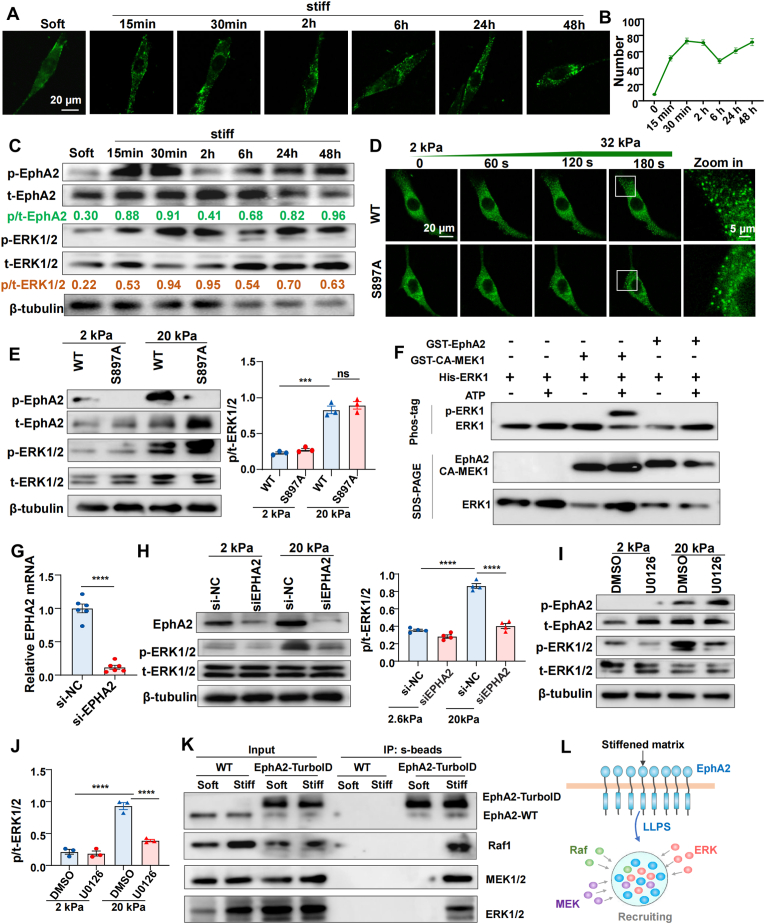
EphA2 condensation facilitate ERK1/2 recruitment and activation by creating a signaling hub. **(A)** Immunofluorescence of EphA2 in HASMCs grown on SPAAC hydrogels with a 4:1 DBCO:N3 stoichiometric ratio stiffened from 2 kPa to 32 kPa for different times. **(B)** Quantification of the number of EphA2 condensates in **(A)**. **(C)** Western blotting to detect the phosphorylation status of EphA2-S897 and ERK1/2 in HASMCs grown on SPAAC hydrogels at different times. **(D)** Live cell imaging of HASMCs transfected with EGFP-EphA2 or EGFP-EphA2-S897A. HASMCs were seeded on SPAAC and stiffened them to E′ = 32 kPa by exposure to 365 nm light. **(E)** Western blotting to detect the phosphorylation status of EphA2-S897 and ERK1/2 in HASMCs seeded on 2 kPa or 20 kPa gels for 24 h, and quantification of p/t-ERK1/2. HASMCs were all transfected with siEPHA2, and then transfected with EphA2-WT or S897A mutant plasmid. n = 3 biological replicates. **(F)** In vitro kinase activity assay. The phosphorylation status of ERK1 was detected by Phos-tag gel. GST-constitutively active-MEK1 (GST-CA-MEK1) was used as a positive control. Protein abundance of GST-EphA2 (561–976 aa), GST-CA-MEK1 and His-ERK1 were shown in SDS-PAGE gel. **(G)** The knockdown efficiency of *EPHA2* targeted siRNA in HASMCs assessed by RT-qPCR. n = 4 biological replicates. **(H)** Left: Western blotting to detect the phosphorylation status of EphA2 and ERK1/2 in HASMCs transfected with scrambled siRNA or *EPHA2* targeted siRNA, and cultured on 2 kPa or 20 kPa gels. Right: semi-quantification of the ERK1/2 phosphorylation level performed by using ImageJ. n = 4 biological replicates. **(I)** Western blotting to detect the phosphorylation status of EphA2 and ERK1/2 in HASMCs seeded on 2 kPa or 20 kPa gels for 24 h. HASMCs were treated with DMSO or MEK inhibitor (U0126, 10 μM). **(J)** Quantification of p/t-ERK1/2 in **(I)**. n = 3 biological replicates. **(K)** TurboID proximity labeling technology to detect the proximity partners of EphA2 in HASMCs seeded on 2 kPa or 20 kPa gels. **(L)** Schematic model illustrating EphA2 condensates act as a signaling hub by recruiting key components of the MAPK pathway to promote ERK1/2 activation. Data were expressed as the means ± SEMs and analyzed by unpaired *t*-test (G) or two-way ANOVA followed by the Tukey's multiple Comparison (E,H and J). ∗∗∗P < 0.001, ∗∗∗∗P < 0.0001.

To elucidate the relationship between EphA2 LLPS and ERK1/2 activation, we first conducted time-course imaging of EphA2 condensate dynamics using in situ stiffening bioclick hydrogels. EphA2 condensates formed rapidly upon matrix stiffening, progressively increased over 30 min, slightly decreased by 2 h, then rebounded and were sustained for up to 48 h (Fig. 4A and B). Given previous reports that matrix stiffness induces phosphorylation of EphA2 at serine 897 (S897) [19,29,30], we asked whether this modification triggers ERK1/2 activation. Western blot analysis revealed similar temporal dynamics for both p-EphA2(S897) and p-ERK1/2, characterized by an initial increase, subsequent decline, and later rebound, though ERK phosphorylation lagged slightly behind EphA2 phosphorylation (Fig. 4C). To test whether S897 phosphorylation is required for ERK activation, we introduced an S897A point mutation. However, this mutation did not impair stiffness-induced EphA2 condensation (Fig. 4D) or ERK1/2 phosphorylation (Fig. 4E). In vitro kinase assay also confirmed that EphA2 does not directly phosphorylate ERK (Fig. 4F).

Notably, EphA2 knockdown markedly suppressed stiffness-induced ERK activation (Fig. 4G and H), and pharmacologic inhibition of MEK1/2 (with U0126) abolished ERK phosphorylation (Fig. 4I and J), suggesting that EphA2 condensation may facilitate ERK activation by recruiting MAPK cascade kinases. To explore the mechanism, we employed proximity labeling [31], a technology for tagging the endogenous interaction partners of specific protein. Interestingly, this approach revealed that under stiff matrix conditions, EphA2 condensates enrich not only ERK1/2 but also RAF1 and MEK1/2 (Fig. 4K and L), thereby facilitating proximal signaling within the MAPK cascade.

Taken together, these findings demonstrate that EphA2 condensation facilitate ERK1/2 recruitment and activation by creating a signaling hub.

### 2.5 EphA2-interfering peptides attenuate droplets formation and ERK1/2 activation

**Fig. 5:**
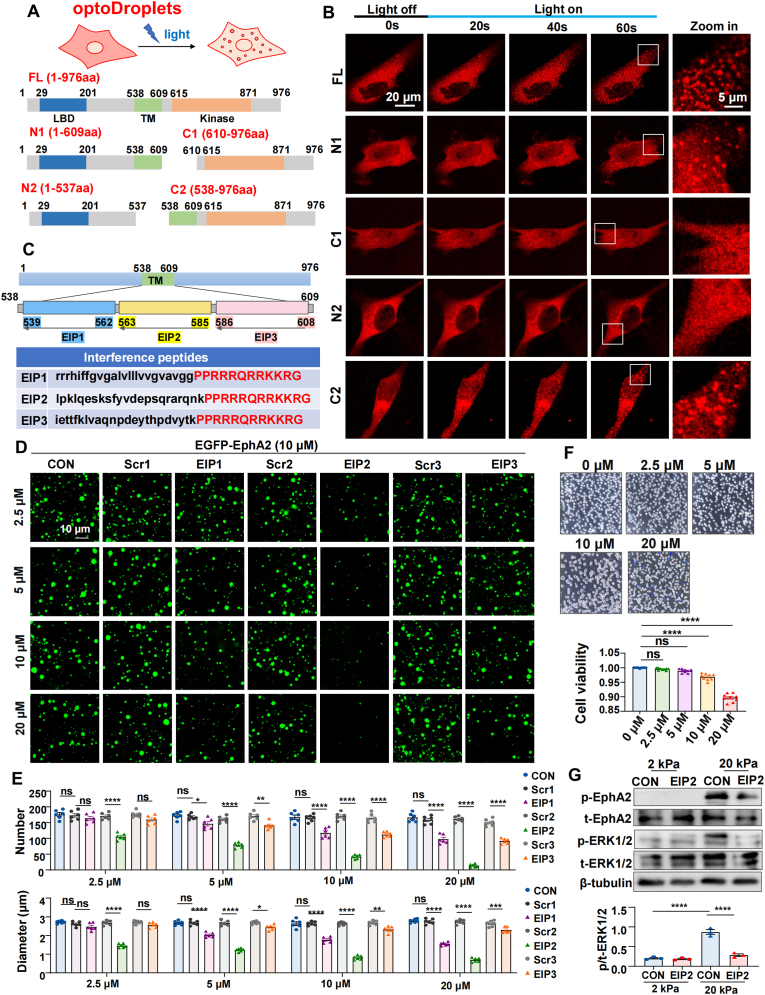
EphA2-Interfering peptides attenuate condensate formation and ERK1/2 activation. **(A)** Schematic diagram of protein domain structure of the full-length EphA2 and the truncated mutations. **(B)** Images of HASMCs expressing Opto-EphA2-FL, Opto-EphA2-N1, Opto-EphA2-C1, Opto-EphA2-N2 or Opto-EphA2-C2 upon blue light exposure. Opto-, the optoDroplet vectors. **(C)** EIP1-EIP3 are designed to block the transmembrane domain of EphA2. The HIV-TAT sequence is in red. **(D)** Droplet formation of EGFP-EphA2 (10 μM) with EIP1-EIP3 and scrambled peptides (10 μM). **(E)** Quantification of the number and diameter of EphA2 droplets in **(D)**. n = 6 biological replicates. The data were analyzed via the Kruskal-Wallis test with Dunn's test. **(F)** Cell viability assessed by Trypan Blue staining in HASMCs treated with EIP2 (10 μM) for 24 h. **(G)** Western blotting to detect the phosphorylation status of EphA2 and ERK1/2 in HASMCs seeded on 2 kPa or 20 kPa gels for 24 h, and treated with EIP2 (10 μM), and quantification of p/t-ERK1/2. n = 3 biological replicates. The data are expressed as the means ± SEMs, and analyzed via one-way ANOVA. (**E** and **F**) and two-way ANOVA followed by Tukey's multiple comparison test (**G**). ∗∗P < 0.01, ∗∗∗P < 0.001, ∗∗∗∗P < 0.0001.

Given the challenge in disrupting the multivalent EphA2-ERK1/2 interface revealed by our structural model (Fig. 3G and H), we aimed to instead inhibit EphA2 LLPS as a strategy to dismantle the downstream signaling hub. To elucidate which domain mediates EphA2 phase separation, we first predicted the intrinsic disordered regions (IDRs) in EphA2 using Disopred3, which revealed the presence of IDRs within the N-terminus, transmembrane domain, and C-terminus of EphA2 (Fig. S7). We then employed an optoDroplet (Opto-) system [32], fusing full-length EphA2 or truncation mutants to the light-sensitive CRY2 oligomerization domain to enable optogenetic control of condensation. The constructs included Opto-full-length (Opto-FL), Opto-N1 (extracellular and transmembrane domains), Opto-C1 (intracellular domain only), Opto-N2 (extracellular domain only) and Opto-C2 (transmembrane and intracellular domains) (Fig. 5A). Upon blue light stimulation, Opto-FL, Opto-N1, and Opto-C2 readily formed droplets, whereas Opto-N2 and Opto-C1 failed to undergo phase separation (Fig. 5B), suggesting that the transmembrane domain is essential for EphA2 droplet formation.

**Fig. 6:**
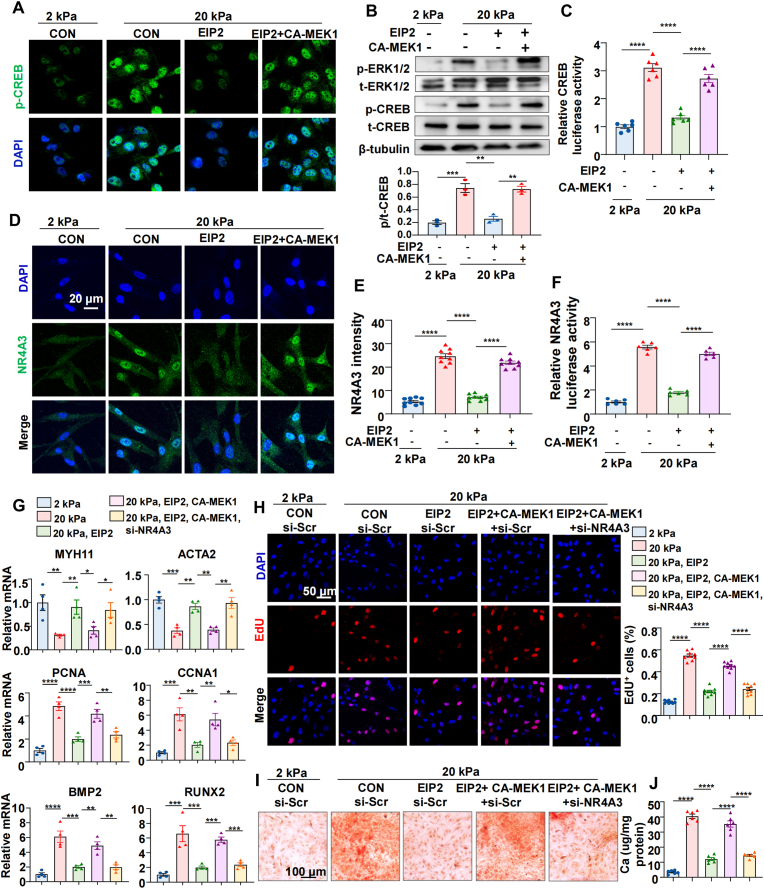
The EphA2-ERK1/2-CREB axis promotes NR4A3 expression and VSMCs proliferation and calcification. **(A)** Immunofluorescence of p-CREB in HASMCs grown on 2 kPa or 20 kPa gels for 24 h. HASMCs were treated with EIP2 (10 μM) and transfected with pcDNA or a constitutively active S218D/S222D MEK1 mutant (CA-MEK1). (B) Western blotting was used to detect the phosphorylation status of ERK1/2 and CREB in HASMCs treated as described in (A). n = 3 biological replicates. **(C)** Detection of CREB transcriptional activity via a luciferase assay in HASMCs treated as described in **(A)**. The luciferase activity was normalized to the β-galactosidase activity. n = 6 biological replicates. **(D)** Immunofluorescence of NR4A3 in HASMCs treated as described in **(A)**. **(E)** Quantification of the immunofluorescence intensity of NR4A3 via ImageJ software. n = 9 images from 3 biological replicates. **(F)** Detection of NR4A3 transcriptional activity via a luciferase assay in HASMCs treated as in **(D)**. n = 6 biological replicates. **(G)** qRT-PCR analysis of VSMC phenotypic marker genes. **(H)** Representative images of EdU-stained HASMCs subjected to indicated treatments, and quantification of EdU-positive cells. **(I)** Alizarin Red staining was performed to assess calcium deposition in HASMCs with indicated treatments. The images reveal the presence of calcified nodules (red staining). **(J)** Quantification of the calcium content in HASMCs via a colorimetric assay. n = 6 biological replicates. The data are expressed as the means ± SEMs and were analyzed via two-way ANOVA followed by Tukey's multiple comparison test. ∗∗P < 0.01, ∗∗∗P < 0.001, ∗∗∗∗P < 0.0001.

We next asked whether targeting specific interaction motifs within these IDRs could disrupt phase separation. We designed a series of cell-permeable EphA2-interfering peptides (EIPs) composed of d-amino acids in retroreversed sequence and fused to the HIV-TAT cell-penetrating motif [33]. These peptides were engineered to bind three distinct surface regions of the transmembrane domain (Fig. 5C). To ensure the specificity and efficacy of the EIPs, scrambled peptides were used as controls. We first assessed the inhibitory activity by treating recombinant EGFP-EphA2 proteins with increasing concentrations of each peptide. In vitro phase separation assays demonstrated a concentration-dependent inhibition, with EIP2 exhibiting the most potent effect, while the scrambled peptides had no effect (Fig. 6D and E). Consistent with these findings, live-cell imaging confirmed that EIP2 significantly suppressed EphA2 droplet formation at concentrations as low as 5 μM (Fig. S8). We next evaluated the cellular tolerability of EIP2. Trypan blue exclusion assays indicated that cell viability remained above 95 % at concentrations up to 10 μM, whereas treatment with 20 μM EIP2 reduced viability to below 90 % (Fig. 5F). We therefore selected 10 μM EIP2 for subsequent cellular experiments. Excitingly, EIP2 significantly inhibited stiffness-induced ERK1/2 phosphorylation (Fig. 5G).

Collectively, these results demonstrate that the transmembrane domain is critical for EphA2 LLPS, and that specifically targeting this region with interfering peptides can effectively attenuate both phase separation and downstream ERK1/2 activation.

### 2.6 The EphA2-ERK1/2-CREB axis promotes NR4A3 expression and VSMCs proliferation and calcification

To identify the transcriptional regulatory network and key transcription factors downstream of EphA2 signaling, we performed RNA-Seq on HASMCs with or without *EPHA2* knockdown under stiff substrates. A total of 2762 genes were significantly down-regulated and 2981 genes were significantly up-regulated (adjusted P-value <0.05, |log_2_FoldChange| > 2) (Fig. S9A). KEGG pathway enrichment analysis revealed that the 2762 down-regulated genes were notably enriched in the MAPK signaling pathway, PI3K-AKT pathway, and Hippo signaling pathway, etc (Fig. S9B). Transcription factor enrichment analysis identified cAMP response element-binding protein (CREB) within the top-10 enriched factors (Fig. S9C). Given that extensive evidence has established that activated ERK1/2 phosphorylates and activates CREB to promote its transcriptional activity [[34], [35], [36]], we investigated whether the EphA2-ERK axis mediates CREB activation. Immunofluorescence staining and Western blot analysis revealed that EIP2 significantly inhibited stiffness-induced CREB phosphorylation and transcriptional activity, whereas it was reversed by a constitutively active MEK1 mutant (CA-MEK1) (Fig. 6A–C).

To identify transcription factors more directly linked to pathological vascular stiffening, we analyzed transcriptomic data from vessels of 5/6 nephrectomy and sham-operated mice (GSE159833). In this disease-relevant context, we identified 693 genes significantly upregulated in stiffened vessels (Fig. S9D). Transcription factor enrichment analysis of this gene set ranked NR4A3 as the most significantly enriched factor (Fig. S9E), nominating it as a key transcriptional regulator driving pathological vascular changes. Notably, CREB is known to regulate multiple downstream genes, including members of the NR4A nuclear receptor family [[37], [38], [39]]. Consistent with this, our transcriptomic analysis revealed that *EPHA2* knockdown significantly suppressed the expression of NR4A family members, particularly NR4A3, along with a cohort of genes associated with cell proliferation and calcification (Fig. S9F). This finding directly links the upstream EphA2-CREB axis activation to a downstream, pathology-associated effector (NR4A3) at the transcriptional level. More excitingly, we found that EIP2 markedly attenuated the upregulation of NR4A3 expression and transcriptional activity, which were reversed by the expression of constitutively active MEK1 (CA-MEK1) (Fig. 6D–F). Functionally, EIP2 effectively inhibited the pathological phenotypic switch of VSMCs induced by matrix stiffness (Fig. 6G), which accompanied by a significant suppression of aberrant proliferation (Fig. 6H), migration (Fig. S10) and calcification (Fig. 6I and J). While the expression of CA-MEK1 restored both proliferation, migration and calcification in EIP2-treated cells, knockdown of the downstream transcription factor NR4A3 (Fig. S11) abolished these restorative effects (Fig. 6G–J and Fig. S10).

Together, these findings demonstrate that matrix stiffness activates EphA2 phase separation to drive ERK1/2-CREB-mediated NR4A3 upregulation, ultimately promoting the pathological phenotypic switch of VSMCs.

### 2.7 NR4A3 is responsible for stiffness-induced EphA2 expression and forms a positive feedback loop

We next asked whether NR4A3, as a key transcriptional output of this pathway, might in turn regulate EphA2 expression itself, thereby forming a feedback loop that could amplify and sustain the stiffnss response. To test this, we first predicted potential NR4A3 binding sites within the EphA2 promoter region using the JASPAR database and identified two high-confidence candidate sites (Fig. S12A). A ChIP assay revealed that NR4A3 can bind to both promoter region 1 (P1) and promoter region 2 (P2), but its binding with P2 is stronger than that with P1 (Fig. S12B). We further confirmed the binding of NR4A3 to the P2 region by knocking down NR4A3 (Fig. S12C and S12D). qRT-PCR revealed that NR4A3 knockdown significantly reduced the increase in EphA2 expression caused by matrix stiffness, indicating that NR4A3 is responsible for stiffness-induced EphA2 expression (Fig. S12E). Notably, increased EphA2 expression and arterial stiffening are not only the consequence of NR4A3 activation but also promote EphA2-ERK1/2-CREB axis activation to form a positive feedback loop (Fig. S12F).

### 2.8 Stiffness-induced EphA2 phase separation is clinically relevant in human vascular diseases

To investigate the relevance of EphA2 LLPS mechanism in the human clinical context, we analyzed arterial sections from three distinct patient cohorts: patients with chronic kidney disease (CKD), elderly patients with abdominal aortic aneurysm (AAA), and patients with advanced carotid atherosclerosis. Immunofluorescence staining demonstrated a marked upregulation of EphA2 condensates in diseased arteries (Fig. S13), providing direct histopathological evidence for EphA2 LLPS in arterial stiffening across distinct disease etiologies.

To further explore the clinical association, we measured circulating EphA2 levels in plasma from control subjects, CKD patients, and atherosclerosis patients, whose baseline characteristics showed no significant differences (Table S1). Patients with CKD and atherosclerosis exhibited significantly elevated plasma EphA2 levels compared to controls (Fig. S14A). More importantly, group-specific correlation analyses within each cohort revealed a consistent positive trend between plasma EphA2 levels and carotid-to-femoral pulse wave velocity (cf-PWV), a direct clinical measure of arterial stiffness (Fig. S14B and S14C).

Collectively, these human data establish that EphA2 LLPS is a histopathological feature of clinical vascular disease and that elevated circulating EphA2 correlates with arterial stiffness severity, thereby strengthening the clinical relevance and generalizability of our mechanistic findings.

### 2.9 Interfering peptides can disrupt EphA2 phase separation and inhibit arterial stiffening

**Fig. 7:**
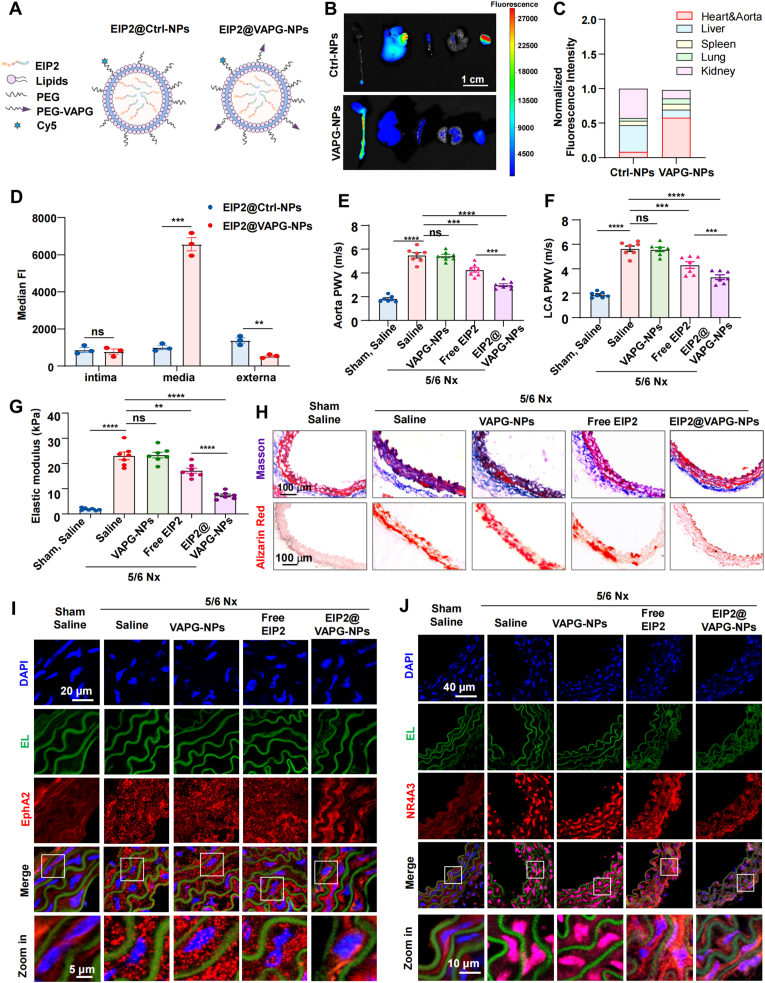
EIP2-NPs targeting EphA2 phase separation inhibit arterial stiffening. (A) Schematic diagram of the EIP2@Ctrl-NPs and EIP2@VAPG-NPs. **(B** and **C)** Ex vivo imaging and fluorescence quantification showing distribution in major organs of 5/6 Nx mice injected with EIP2@Ctrl-NPs or EIP2@VAPG-NPs. **(D)** Flow cytometry quantification of nanoparticle enrichment in the vascular intima (endothelial cells), media (smooth muscle cells), and adventitia (fibroblasts). **(E** and **F)** Pulse wave velocity (PWV) of the aorta and left carotid artery in the indicated mice. Sham and 5/6 Nx mice subjected to tail vein injection of normal saline, empty VAPG-NPs, free EIP2, or EIP2@VAPG-NPs every three days. n = 7 mice. **(G)** The elastic modulus of thoracic aortas from the indicated mice was measured by nanoindentation. n = 7 mice. **(H)** Masson and Alizarin red S staining of the thoracic aortas of the indicated mice. **(I)** Immunofluorescence of EphA2 in the indicated aortas. **(J)** Immunofluorescence of NR4A3 in the indicated aorta. The data are expressed as the means ± SEMs and were analyzed via unpaired *t*-test (D) or two-way ANOVA followed by Tukey's multiple comparison test (E-G). ∗∗P < 0.01, ∗∗∗P < 0.001, ∗∗∗∗P < 0.0001.

To achieve in vivo intervention of EphA2 phase separation, lipid nanoparticles (NPs) were utilized to encapsulate EIP2, thereby enhancing their circulatory persistenc. For active targeting of vascular smooth muscle cells (VSMCs), the nanoparticles (NPs) were functionalized with a lipid-PEG conjugate functionalized with elastin-binding peptide VAPG (Fig. 7A), which specifically recognizes the elastin-rich tunica media [40,41]. To assess the targeting efficacy of VAPG modification toward VSMCs, we first performed in vitro cell experiments. Results showed that VSMCs took up significantly more VAPG-NPs than endothelial cells or fibroblasts. In contrast, Ctrl-NPs were predominantly internalized by endothelial cells and showed low uptake in both VSMCs and fibroblasts (Fig. S15). Subsequently, we injected 5/6 nephrectomized (Nx) mice with either EIP2@Ctrl-NPs or EIP2@VAPG-NPs. After 12 h, in vivo fluorescence imaging revealed stronger accumulation of EIP2@VAPG-NPs in arterial vessels compared to EIP2@Ctrl-NPs (Fig. 7B and C). Further analysis by flow cytometry of isolated aortas showed that VAPG-NPs were significantly enriched in VSMCs, whereas Ctrl-NPs failed to efficiently target smooth muscle cells (Fig. 7D). These results demonstrate that VAPG-modified NPs can effectively deliver drugs to VSMCs.

To evaluate the therapeutic potential of EIP2 in vivo, the 5/6 Nx mice were administered empty VAPG-NPs, free EIP2, or EIP2@VAPG-NPs every three days. No significant differences in body weight were observed among the Nx groups (Fig. S16A). BUN and serum creatinine levels were markedly elevated in all Nx mice regardless of treatment, indicating that neither NP encapsulation nor EIP2 administration ameliorated renal failure (Fig. S16B and S16C). However, both free EIP2 and EIP2@VAPG-NPs slightly reduced the blood pressure of 5/6 Nx mice (Fig. S16D and S16E), suggesting that they may have improved vascular compliance.

Notably, although free EIP2 partially attenuated the chronic kidney failure-induced increases in PWV (Fig. 7E and F), aortic elastic modulus (Fig. 7G), and collagen and calcium deposition (Fig. 7H), EIP2@VAPG-NPs exhibited significantly stronger therapeutic efficacy than free EIP2 (Fig. 7E–H). What's more, both EphA2 condensates and NR4A3 expression were significantly elevated in Nx arteries. Free EIP2 partially suppressed EphA2 droplet formation and NR4A3 expression, and EIP2@VAPG-NPs showed markedly stronger inhibition compared to free EIP2 (Fig. 7I–J, Fig. S17).

Altogether, the VAPG-conjugated nanoparticle system significantly enhances the targeted delivery of EIP2 to vascular smooth muscle cells, leading to superior therapeutic outcomes in attenuating vascular stiffening.

## 3 Discussion

LLPS has emerged as a critical regulatory mechanism in cellular biology, enabling specific molecules to form membraneless organelles and regulate cellular processes [20]. While LLPS has been extensively studied in the context of neurodegenerative diseases and cancer [42], its role in cardiovascular diseases remains largely unknown. In this study, we identified EphA2, a receptor tyrosine kinase, as a key mechanosensitive regulator of arterial stiffening through its ability to undergo LLPS in response to matrix stiffness. Our findings reveal a novel EphA2-ERK-NR4A3 signaling axis that drives vascular remodeling and arterial stiffening, providing new insights into the pathogenesis of cardiovascular diseases. On the one hand, substrate stiffness induces EphA2 phase separation and activation; on the other hand, activated EphA2 further promotes ERK1/2-NR4A3 signaling, leading to SMC proliferation and calcification [25,43,44]. This bidirectional relationship suggests that EphA2 not only responds to but also amplifies arterial stiffness.

The discovery of EphA2 LLPS as a mechanosensitive process adds a new dimension to our understanding of how mechanical cues are transduced into biochemical signals in VSMCs. Like other mechanoreceptors, such as DDR1 [13]. EphA2 forms condensates that exhibit characteristic liquid-like behaviors—including spherical morphology, fluidity, fusion, and fission—supporting its role as a regulatory hub in mechanotransduction. By concentrating key signaling components, EphA2 LLPS facilitates their activation and enhances downstream signaling amplification. Our results are consistent with prior studies demonstrating that LLPS can promote localized biomolecular concentration to accelerate reaction kinetics [20,45,46], reinforcing the notion that EphA2 condensates function as critical amplifiers of mechanosensitive pathways. However, whether EphA2 serves as a primary mechanosensor that responds directly to membrane tension or whether its condensation is triggered upstream by other mechanosensing elements remains unknown. Future studies employing direct force manipulation tools, such as magnetic tweezers, could help delineate the precise hierarchical sequence of these mechanosensing events. Moreover, while our work establishes EphA2 LLPS as an essential signaling hub, the exact molecular triggers initiating its condensation require further clarification. For example, our co-immunoprecipitation and mass spectrometry data revealed several heat shock proteins uniquely enriched under stiff matrix conditions, suggesting that these chaperones may act as important co-factors promoting EphA2 condensate assembly. Integrating structural analyses with targeted validation of such candidate co-factors will be crucial for a complete mechanistic understanding.

The involvement of the ERK1/2-CREB-NR4A3 axis downstream of EphA2 LLPS provides a mechanistic link between matrix stiffness and vascular remodeling. ERK1/2 activation has been previously implicated in cell proliferation and osteogenic differentiation [3,23,47,48], both of which contribute to arterial stiffening. Although the link between matrix stiffness and ERK activation has been described in many studies, the mechanoreceptors upstream of matrix stiffness-induced ERK activation are still not fully understood. By demonstrating that EphA2 LLPS drives ERK1/2 activation and subsequent NR4A3 expression, we revealed a novel pathway through which mechanical stimuli are translated into transcriptional changes that promote vascular pathology. The positive feedback loop between NR4A3 and EphA2 further underscores the self-amplifying nature of this signaling cascade, suggesting that targeting EphA2 LLPS could disrupt this cycle and provide therapeutic benefits.

Our findings also highlight the therapeutic potential of targeting EphA2 LLPS. Although previous studies have reported that the small-molecule inhibitor ALW-II-41-27 can inhibit EphA2 phase separation [22], it can nonspecifically inhibit many Eph receptors and is not LLPS specific [49]. In contrast, we developed an interfering peptide that selectively targets the EphA2 phase separation domain. Delivered in vivo via VAPG-modified nanoparticles, this peptide effectively disrupted EphA2 condensates in VSMCs and attenuated arterial stiffening, demonstrating the feasibility of LLPS-specific pharmacological intervention. Notably, beyond rodent models, we observed preliminary evidence of EphA2 condensate formation in human vascular specimens—including tissues from patients with chronic kidney disease, abdominal aortic aneurysm, and advanced carotid atherosclerosis. Although this suggests that EphA2 LLPS may be a conserved mechanism across human vascular pathologies such as vascular aging and atherosclerosis, its functional causality and clinical relevance in these specific contexts remain to be established. To advance the translational potential of this mechano-targeted strategy, future studies should systematically evaluate its efficacy across diverse vascular diseases and explore its therapeutic benefit, either alone or in combination with other emerging mechano-modulatory approaches.

In conclusion, this study elucidates a novel mechanotransduction pathway in which EphA2 LLPS drives arterial stiffening through the ERK1/2-CREB-NR4A3 axis. Our findings not only expand the understanding of LLPS in cardiovascular diseases but also provide a potential therapeutic target for arterial stiffening.

## CRediT authorship contribution statement

**Jia-Yu Liu:** Writing – original draft, Methodology, Investigation, Funding acquisition, Formal analysis, Data curation, Conceptualization. **Geng Shen:** Investigation, Formal analysis, Data curation. **Yi-Chen Lin:** Resources, Investigation. **Jing Chen:** Data curation. **Qin-Ye Chen:** Data curation. **Mo-Jun Lin:** Writing – review & editing, Visualization, Supervision, Conceptualization.

## Ethics approval and consent to participate

All procedures involving human samples were performed in accordance with the ethical standards of the Medical Ethics Committee of the First Affiliated Hospital of Fujian Medical University (Approval Nos. [2025]216 and [2023]508). Informed consent was obtained from all participants. The animal study was reviewed and approved by the Animal Care and Use Committee of Fujian Medical University and approved by the Ethics Committee of Fujian Medical University (IACUC FJMU 2025-0224).

## Sources of funding

This study was funded by the National Natural Science Foundation of China (#82500495 to Jia-Yu Liu, #32571312 to Mo-Jun Lin), the Natural Science Foundation of Fujian Province (2025J01680 to Jia-Yu Liu), the Educational and Scientific Research Projects for Young and Middle-aged Teachers in Fujian Province (JZ240021 to Jia-Yu Liu) and the Startup Fund for High-level Talents of Fujian Medical University (XRCZX2024023 to Jia-Yu Liu).

## Declaration of competing interest

The authors declare that they have no competing interests.

## Data Availability

The detailed Materials and Methods section is available in the Expanded Methods. All primers used are listed in the Supplementary Tables. All data related to the findings of this study are available from the corresponding author upon reasonable request.

## References

[1] Vatner S.F., Zhang J., Vyzas C., Mishra K., Graham R.M., Vatner D.E. (2021). Vascular stiffness in aging and disease. Front. Physiol..

[2] Boutouyrie P., Chowienczyk P., Humphrey J.D., Mitchell G.F. (2021). Arterial stiffness and cardiovascular risk in hypertension. Circ. Res..

[3] Wang J., Xie S.A., Li N., Zhang T., Yao W., Zhao H. (2022). Matrix stiffness exacerbates the proinflammatory responses of vascular smooth muscle cell through the DDR1-DNMT1 mechanotransduction axis. Bioact. Mater..

[4] Xie S.A., Zhang T., Wang J., Zhao F., Zhang Y.P., Yao W.J. (2018). Matrix stiffness determines the phenotype of vascular smooth muscle cell in vitro and in vivo: role of DNA methyltransferase 1. Biomaterials.

[5] Swiatlowska P., Sit B., Feng Z., Marhuenda E., Xanthis I., Zingaro S. (2022). Pressure and stiffness sensing together regulate vascular smooth muscle cell phenotype switching. Sci. Adv..

[6] Abdulhussein R., Koo D.H., Vogel W.F. (2008). Identification of disulfide-linked dimers of the receptor tyrosine kinase DDR1. J. Biol. Chem..

[7] Davis M.J., Earley S., Li Y.S., Chien S. (2023). Vascular mechanotransduction. Physiol. Rev..

[8] Zhang L., Zhou J., Kong W. (2025). Extracellular matrix in vascular homeostasis and disease. Nat. Rev. Cardiol..

[9] Du Z.F., Lovly C.M. (2018). Mechanisms of receptor tyrosine kinase activation in cancer. Mol. Cancer.

[10] Lemmon M.A., Schlessinger J. (2010). Cell signaling by receptor tyrosine kinases. Cell.

[11] Bian J.S., Chen J., Zhang J., Tan J., Chen Y., Yang X. (2024). ErbB3 governs endothelial dysfunction in hypoxia-induced pulmonary hypertension. Circulation.

[12] Cheng Q., Bilgin C.C., Fontenay G., Chang H., Henderson M., Han J. (2016). Stiffness of the microenvironment upregulates ERBB2 expression in 3D cultures of MCF10A within the range of mammographic density. Sci. Rep..

[13] Liu J., Wang J., Liu Y., Xie S.A., Zhang J., Zhao C. (2023). Liquid-Liquid phase separation of DDR1 counteracts the Hippo pathway to orchestrate arterial stiffening. Circ. Res..

[14] Liu J.Y., Zhao C.R., Xiao X., Li A.H., Liu Y.Q., Zhao J.N. (2023). Endothelial discoidin domain receptor 1 senses flow to modulate YAP activation. Nat. Commun..

[15] Liang L.Y., Patel O., Janes P.W., Murphy J.M., Lucet I.S. (2019). Eph receptor signalling: from catalytic to non-catalytic functions. Oncogene.

[16] Zhu Y., Su S.A., Shen J., Ma H., Le J., Xie Y. (2024). Recent advances of the Ephrin and Eph family in cardiovascular development and pathologies. iScience.

[17] Zeng J., Wu Q., Xiong S., Lu C., Zhang Z., Huang H. (2023). Inhibition of EphA2 protects against atherosclerosis by synergizing with statins to mitigate macrophage inflammation. Biomed. Pharmacother..

[18] Finney A.C., Funk S.D., Green J.M., Yurdagul A., Rana M.A., Pistorius R. (2017). EphA2 expression regulates inflammation and fibroproliferative remodeling in atherosclerosis. Circulation.

[19] Fattet L., Jung H.Y., Matsumoto M.W., Aubol B.E., Kumar A., Adams J.A. (2020). Matrix rigidity controls epithelial-mesenchymal plasticity and tumor metastasis via a mechanoresponsive EPHA2/LYN complex. Dev. Cell.

[20] Banani S.F., Lee H.O., Hyman A.A., Rosen M.K. (2017). Biomolecular condensates: organizers of cellular biochemistry. Nat. Rev. Mol. Cell Biol..

[21] Banani S.F., Rice A.M., Peeples W.B., Lin Y., Jain S., Parker R. (2016). Compositional control of phase-separated cellular bodies. Cell.

[22] Li Y., Peng Q., Wang L. (2023). EphA2 as a phase separation protein associated with ferroptosis and immune cell infiltration in colorectal cancer. Aging (Albany NY).

[23] Lavoie H., Gagnon J., Therrien M. (2020). ERK signalling: a master regulator of cell behaviour, life and fate. Nat. Rev. Mol. Cell Biol..

[24] Rius J., Martinez-Gonzalez J., Crespo J., Badimon L. (2004). Involvement of neuron-derived orphan receptor-1 (NOR-1) in LDL-induced mitogenic stimulus in vascular smooth muscle cells: role of CREB. Arterioscler. Thromb. Vasc. Biol..

[25] Martinez-Gonzalez J., Canes L., Alonso J., Ballester-Servera C., Rodriguez-Sinovas A., Corrales I. (2021). NR4A3: a key nuclear receptor in vascular biology, cardiovascular remodeling, and beyond. Int. J. Mol. Sci..

[26] Han Y., Zhang J., Huang S., Cheng N., Zhang C., Li Y. (2021). MicroRNA-223-3p inhibits vascular calcification and the osteogenic switch of vascular smooth muscle cells. J. Biol. Chem..

[27] Yuan Z., Li Y., Zhang S., Wang X., Dou H., Yu X. (2023). Extracellular matrix remodeling in tumor progression and immune escape: from mechanisms to treatments. Mol. Cancer.

[28] Silver J.S., Gunay K.A., Cutler A.A., Vogler T.O., Brown T.E., Pawlikowski B.T. (2021). Injury-mediated stiffening persistently activates muscle stem cells through YAP and TAZ mechanotransduction. Sci. Adv..

[29] Li J.Y., Xiao T., Yi H.M., Yi H., Feng J., Zhu J.F. (2019). S897 phosphorylation of EphA2 is indispensable for EphA2-dependent nasopharyngeal carcinoma cell invasion, metastasis and stem properties. Cancer Lett..

[30] Zhou Y., Sakurai H. (2017). Emerging and diverse functions of the EphA2 noncanonical pathway in cancer progression. Biol. Pharm. Bull..

[31] Qin W., Cho K.F., Cavanagh P.E., Ting A.Y. (2021). Deciphering molecular interactions by proximity labeling. Nat. Methods.

[32] Shin Y., Berry J., Pannucci N., Haataja M.P., Toettcher J.E., Brangwynne C.P. (2017). Spatiotemporal control of intracellular phase transitions using light-activated optoDroplets. Cell.

[33] Xie F., Zhou X., Ran Y., Li R., Zou J., Wan S. (2025). Targeting FOXM1 condensates reduces breast tumour growth and metastasis. Nature.

[34] Xing J., Ginty D.D., Greenberg M.E. (1996). Coupling of the RAS-MAPK pathway to gene activation by RSK2, a growth factor-regulated CREB kinase. Science..

[35] Li L., Fan D., Wang C., Wang J.Y., Cui X.B., Wu D. (2011). Angiotensin II increases periostin expression via Ras/p38 MAPK/CREB and ERK1/2/TGF-beta1 pathways in cardiac fibroblasts. Cardiovasc. Res..

[36] Peng Y., Xiong R., Wang B., Chen X., Ning Y., Zhao Y. (2024). The essential role of angiogenesis in adenosine 2A receptor deficiency-mediated impairment of wound healing involving c-Ski via the ERK/CREB pathways. Int. J. Biol. Sci..

[37] Volakakis N., Kadkhodaei B., Joodmardi E., Wallis K., Panman L., Silvaggi J. (2010). NR4A orphan nuclear receptors as mediators of CREB-dependent neuroprotection. Proc. Natl. Acad. Sci. U. S. A.

[38] Crespo J., Martinez-Gonzalez J., Rius J., Badimon L. (2005). Simvastatin inhibits NOR-1 expression induced by hyperlipemia by interfering with CREB activation. Cardiovasc. Res..

[39] Dou X., Zhu Z., Chen Q., Lu Y. (2025). Sirt1-Mediated transcriptional inhibition of Nr4a3 Alleviates severe acute pancreatitis-associated acute Lung injury. FASEB J..

[40] Puhl D.L., Mohanraj D., Nelson D.W., Gilbert R.J. (2022). Designing electrospun fiber platforms for efficient delivery of genetic material and genome editing tools. Adv. Drug Deliv. Rev..

[41] Xu H., Li S., Liu Y.S. (2022). Nanoparticles in the diagnosis and treatment of vascular aging and related diseases. Signal Transduct. Targeted Ther..

[42] Tsang B., Pritisanac I., Scherer S.W., Moses A.M., Forman-Kay J.D. (2020). Phase separation as a missing mechanism for interpretation of disease mutations. Cell.

[43] Ballester-Servera C., Canes L., Alonso J., Puertas L., Tauron M., Rodriguez C. (2022). Nuclear receptor NOR-1 (Neuron-derived Orphan Receptor-1) in pathological vascular remodelling and vascular remodelling. Clín. Invest. Arterioscler..

[44] Ma W., Jia K., Cheng H., Xu H., Li Z., Zhang H. (2024). Orphan Nuclear Receptor NR4A3 promotes vascular calcification via histone lactylation. Circ. Res..

[45] Zhang H., Ji X., Li P., Liu C., Lou J., Wang Z. (2020). Liquid-liquid phase separation in biology: mechanisms, physiological functions and human diseases. Sci. China Life Sci..

[46] Wang B., Zhang L., Dai T., Qin Z., Lu H., Zhang L. (2021). Liquid-liquid phase separation in human health and diseases. Signal Transduct. Targeted Ther..

[47] Wan W., Cheng B., Zhang C., Ma Y., Li A., Xu F. (2019). Synergistic effect of matrix stiffness and inflammatory factors on osteogenic differentiation of MSC. Biophys. J..

[48] Hwang J.H., Byun M.R., Kim A.R., Kim K.M., Cho H.J., Lee Y.H. (2015). Extracellular Matrix stiffness regulates osteogenic differentiation through MAPK activation. PLoS One.

[49] Choi Y., Syeda F., Walker J.R., Finerty P.J., Cuerrier D., Wojciechowski A. (2009). Discovery and structural analysis of Eph receptor tyrosine kinase inhibitors. Bioorg. Med. Chem. Lett.

