# Supplementary material for: Targeting mechanosensitive EphA2 phase separation to alleviate arterial stiffening
Source: Bioact Mater. 2026 Jan 24;60:203–15. doi: 10.1016/j.bioactmat.2026.01.020 (PMC12860789; doi:10.1016/j.bioactmat.2026.01.020)
Supplement: Multimedia component 1 [file mmc1.pdf]

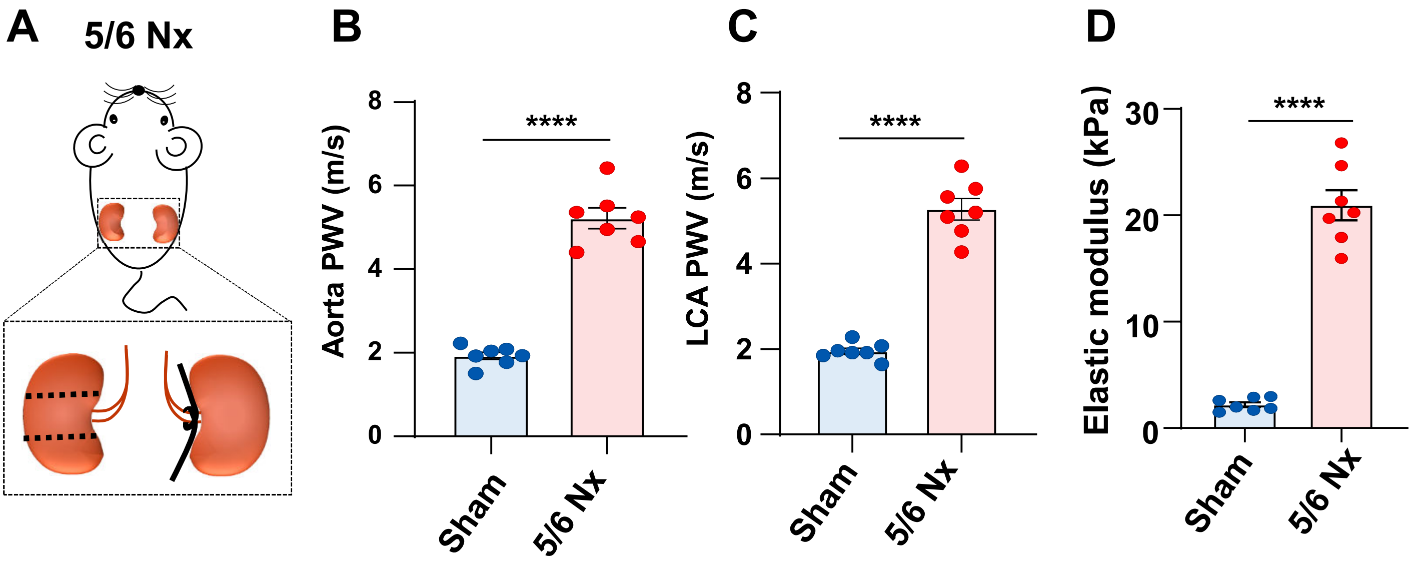

Figure S1. 5/6 nephrectomy induces systemic arterial stiffening in mice.

(A) Schematic showing 5/6 nephrectomy (Nx) surgery in mice. **(B)** and **(C)** Pulse wave velocity (PWV) of the aorta and left carotid artery (LCA) in the sham and 5/6 Nx mice was assessed by ultrasound. n = 7 mice. **(D)** The elastic modulus of thoracic aortas from the sham and 5/6 Nx groups was measured by nanoindentation. n = 7 mice.

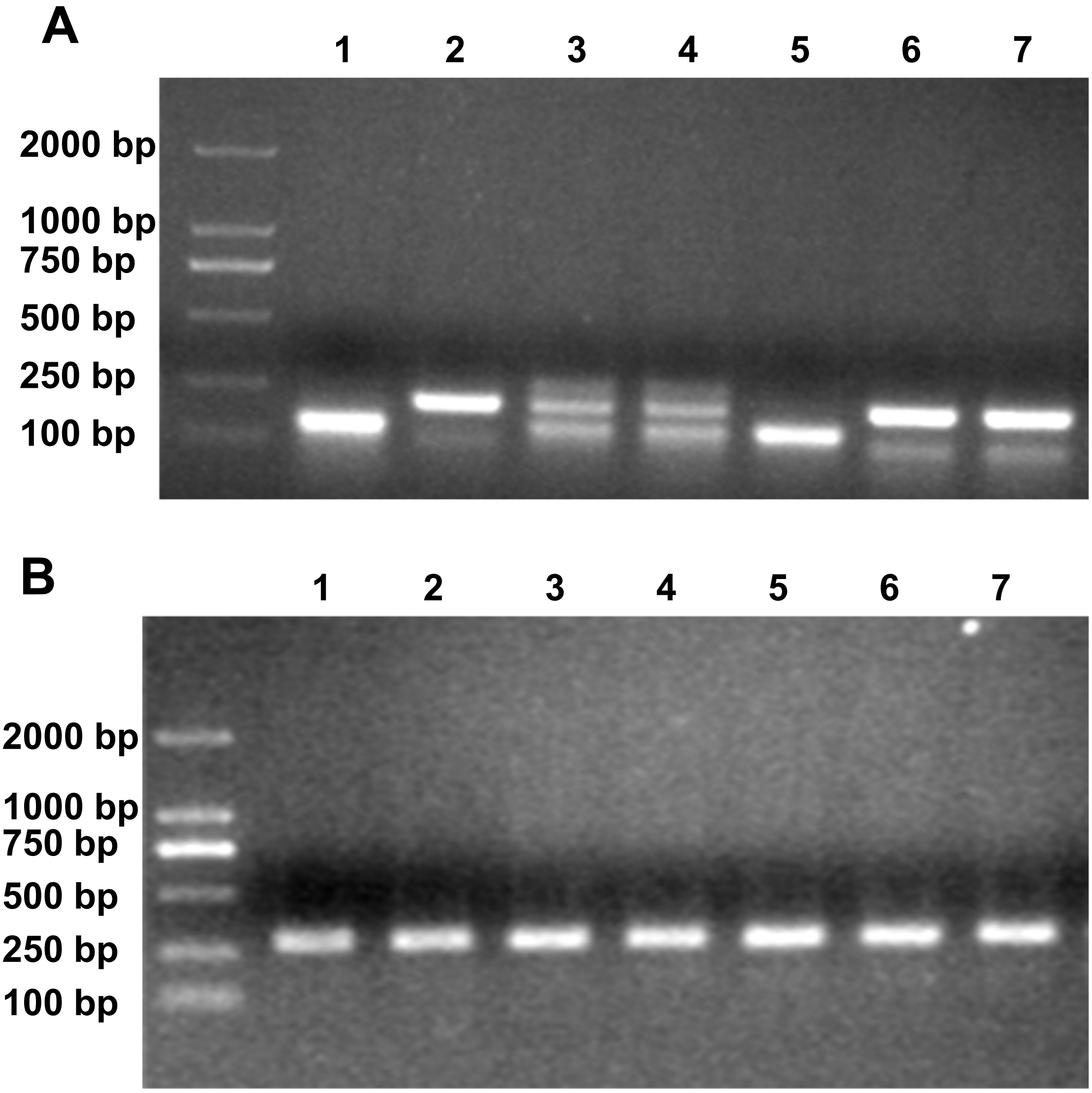

Figure S2. Genotyping of Epha2 knockout mice.

(A) Genotyping of Epha2 flox/flox mice. Among them, 1 and 5 are WT, 2, 6, and 7 are homozygous (Epha2 flox/flox), and 3 and 4 are heterozygous (Epha2 flox/+). **(B)** Genotyping of Myh11-Cre. All mice are Myh11-Cre^{+}.

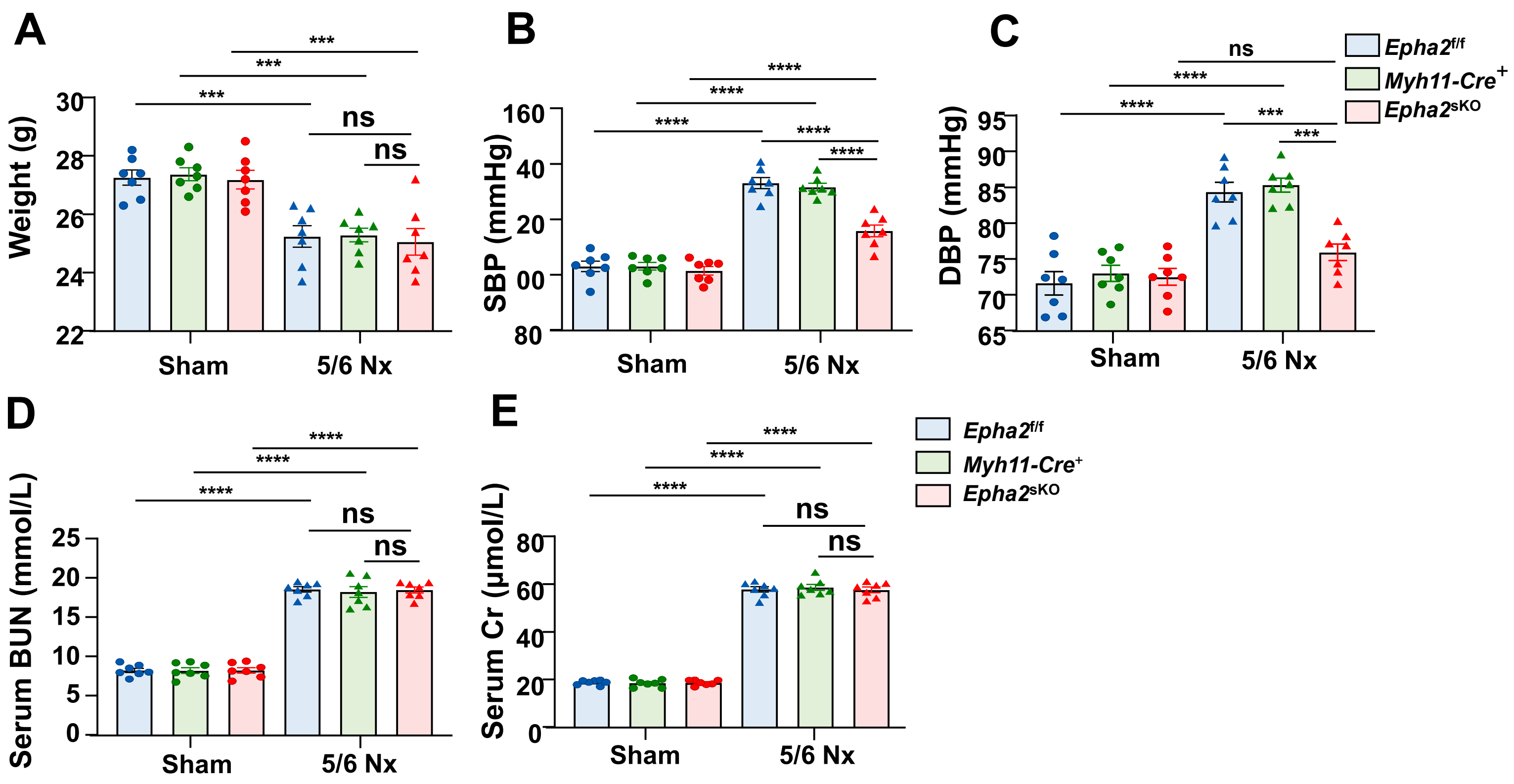

Figure S3. Physiological and biochemical indicators of the Epha2$^{f/f}$, Myh11-CreERT2^{+} and Epha2^{sKO} mice subjected to 5/6 Nx or sham operation. (A) Body weight of mice from the indicated groups. **(B)** and **(C)** Systolic blood pressure (SBP) and diastolic blood pressure (DBP) of mice from the indicated groups. n = 7 mice. **(D)** Serum level of blood urea nitrogen and **(E)** creatinine of mice from the indicated groups. n = 7 mice. Data were expressed as the means ± SEM and analyzed by two-way ANOVA followed by the Tukey's multiple Comparison. BUN, blood urea nitrogen; Cr, creatinine.

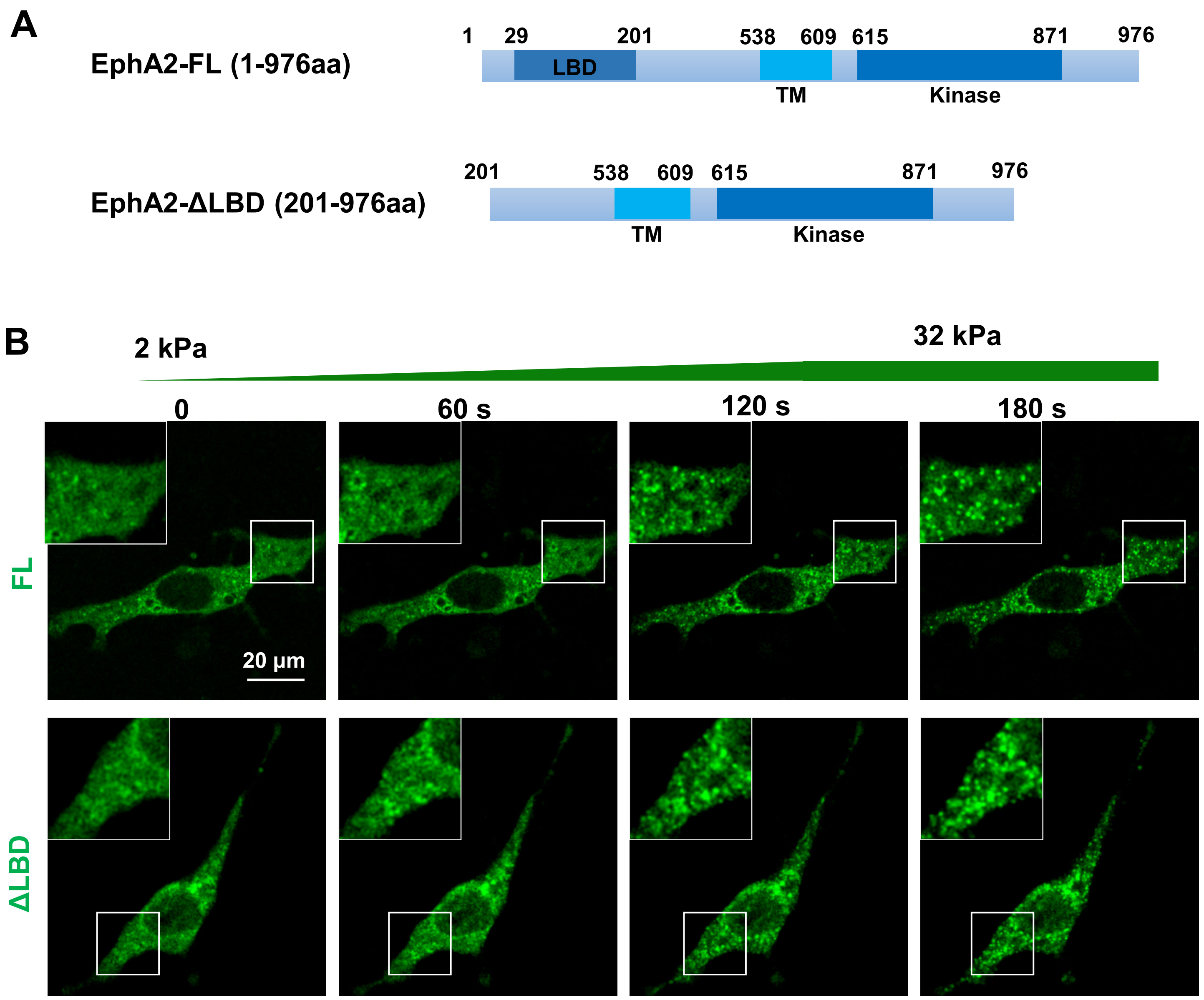

Figure S4. Matrix stiffness induced EphA2 phase separation is independent of its ligand-binding domain.

(A) Schematic diagram of protein domain structure of the full-length EphA2 and EphA2-ΔLBD. **(B)** Live cell imaging of HASMCs transfected with EGFP-EphA2 or EGFP-EphA2-ΔLBD. HASMCs were seeded on strain-promoted alkyne-azide cycloaddition (SPAAC) hydrogels with a 4:1 DBCO:N3 stoichiometric ratio (E′ = 2 kPa) and stiffened them to E′ = 32 kPa by exposure to light (365 nm, 10 mW/cm^2, 120 s). LBD: Ligand-binding domain.

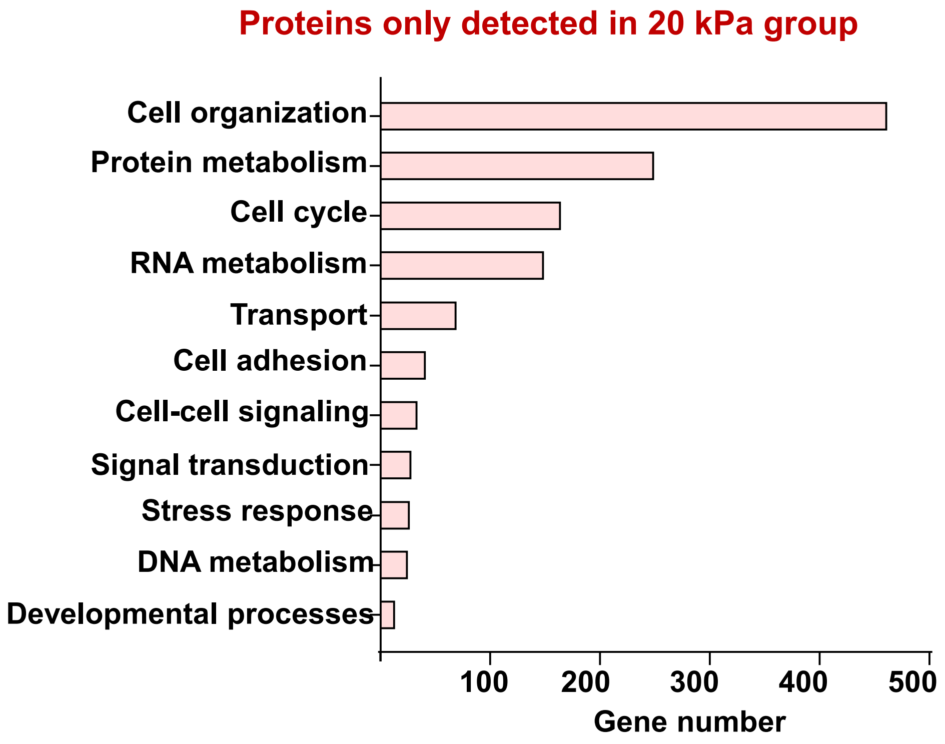

Figure S5. Gene Ontology analysis of proteins uniquely detected in the 20 kPa group. Bar chart shows enriched Biological Process terms from the set of 1,584 proteins identified exclusively under 20 kPa matrix stiffness. The results indicate a predominant involvement of these proteins in processes related to cell organization, protein metabolism, the cell cycle, etc.

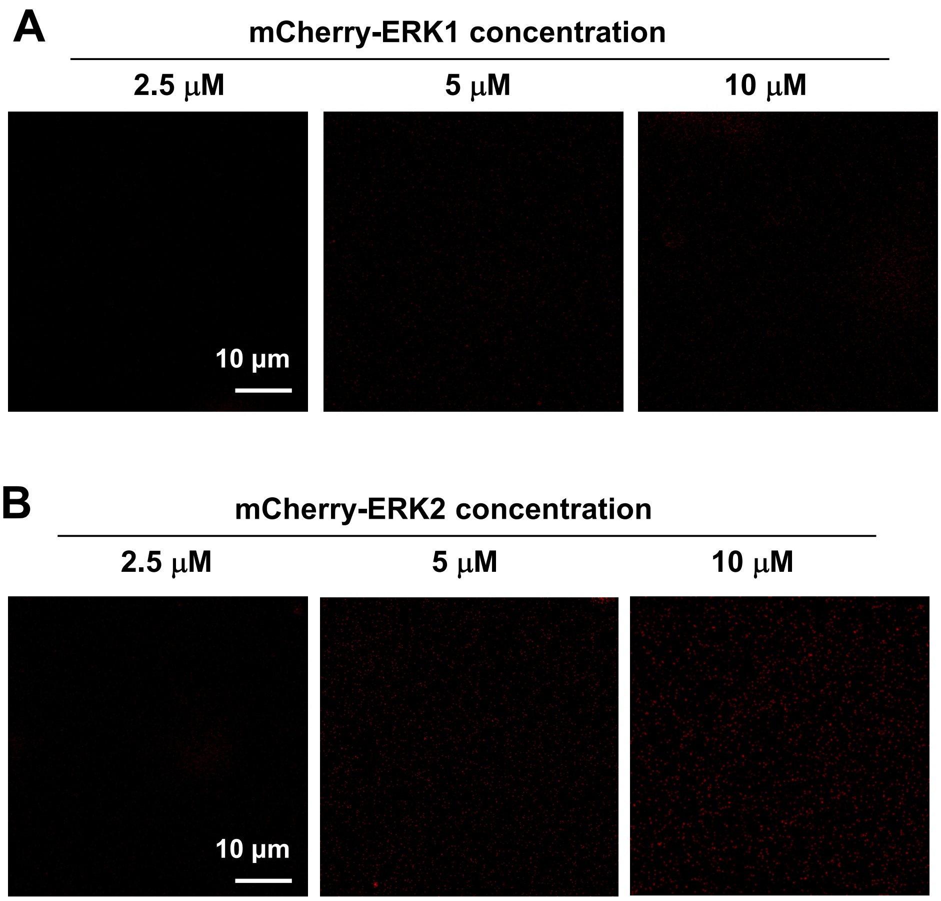

Figure S6. ERK1/2 recombinant proteins cannot form droplets in cell-free system.

(A) Representative fluorescence microscopy images of mCherry-ERK1 solution at different concentrations. **(B)** Representative fluorescence microscopy images of mCherry-ERK2 solution at different concentrations.

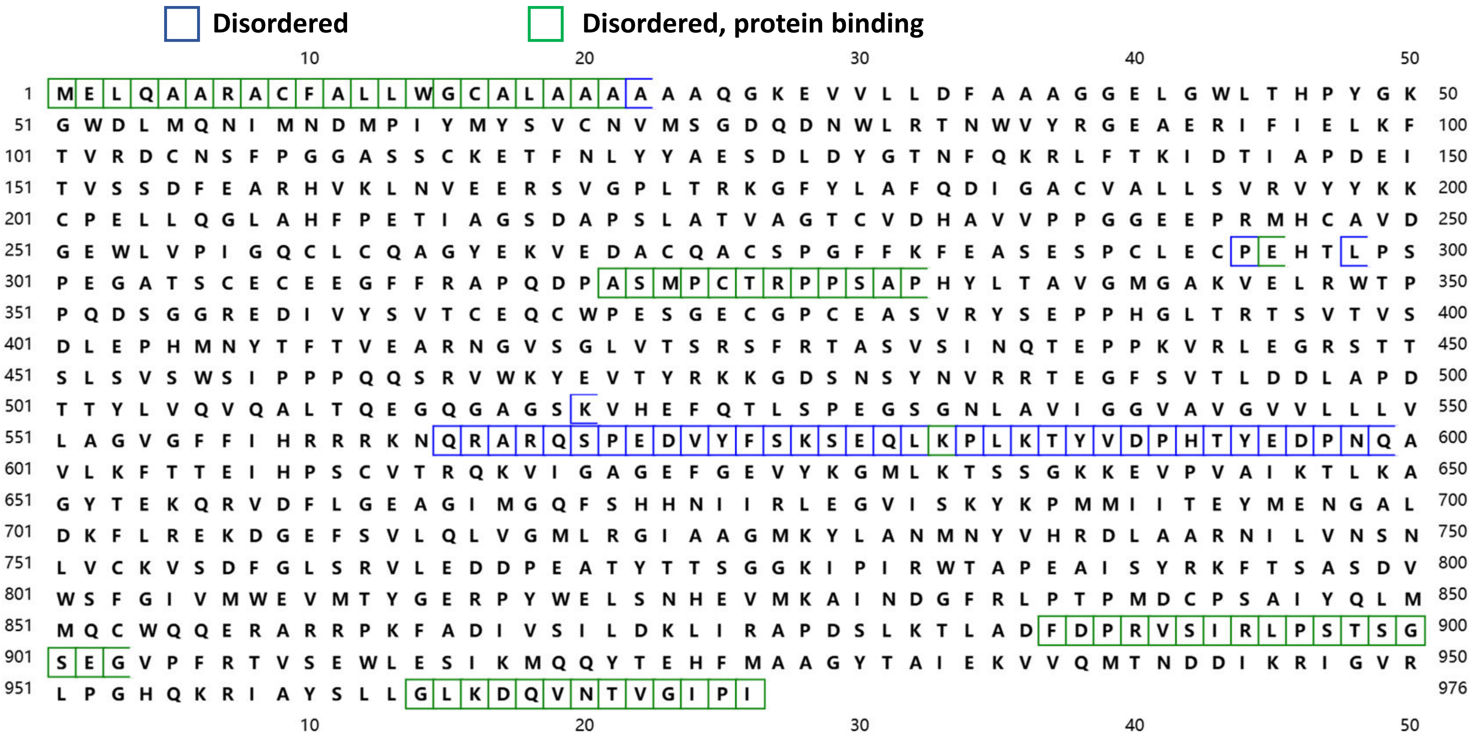

Figure S7. Intrinsically disordered regions prediction of EphA2 by DISOPRED3.

The amino acid sequence of human EphA2 was analyzed using the DISOPRED3 algorithm to predict intrinsically disordered regions. The analysis reveals that EphA2 contains extensive disordered regions, such as the N-terminal domain, transmembrane helix, and the C-terminal cytoplasmic tail.

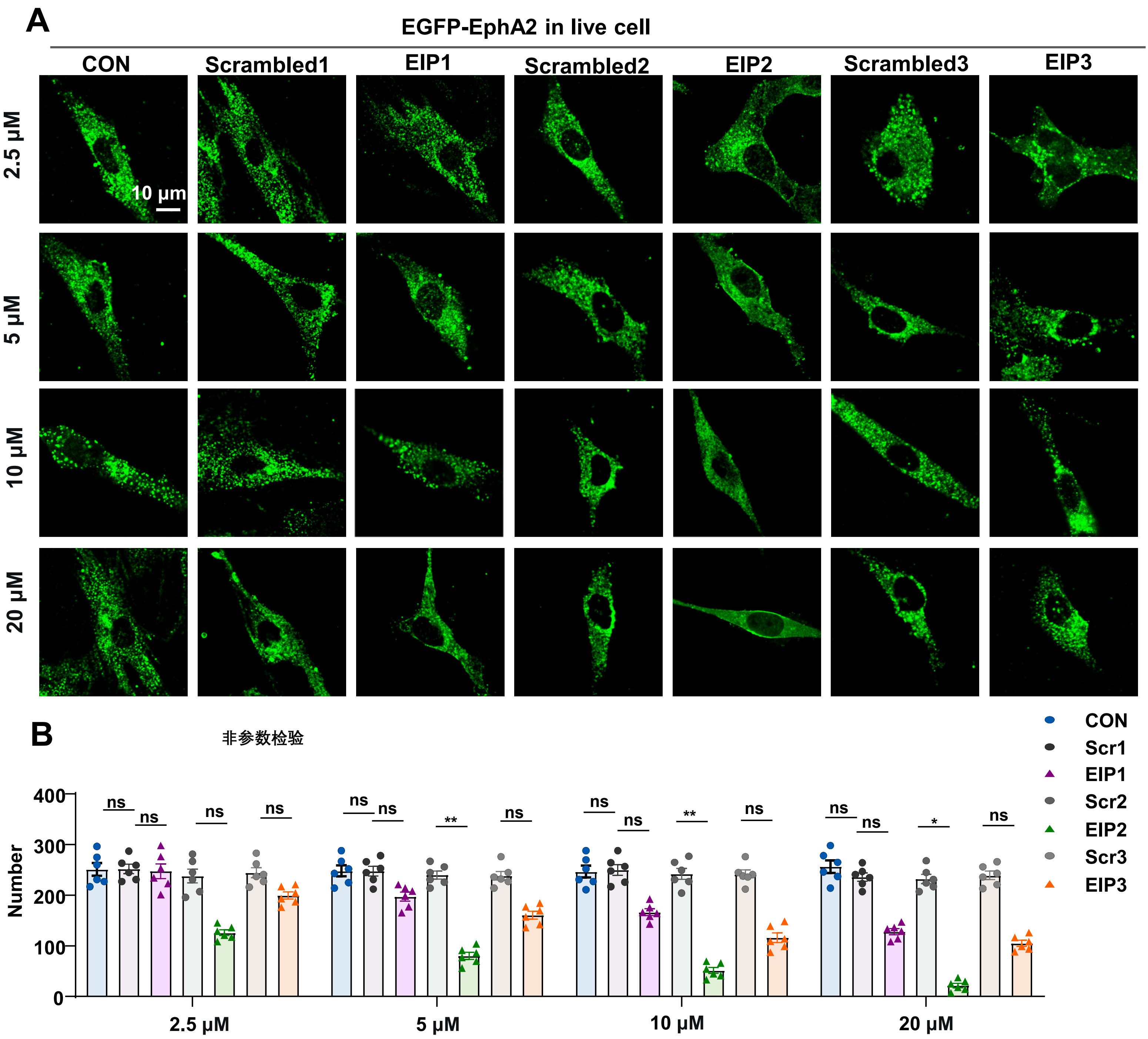

Figure S8. EIP2 effectively inhibits EphA2 droplet formation in HASMCs.

(A) Representative fluorescence microscopy images of HASMCs transfected with EGFP-EphA2. Cells were seeded on 20 kPa gels and treated with varying concentrations of scrambled peptides (Scr) or EIP1–EIP3 for 24 h. **(B)** Quantification of the number of EphA2 droplets per cell from images in (A). n= 6 cells from 3 biological replicates. The data were analyzed via the Kruskal-Wallis test with Dunn's test.

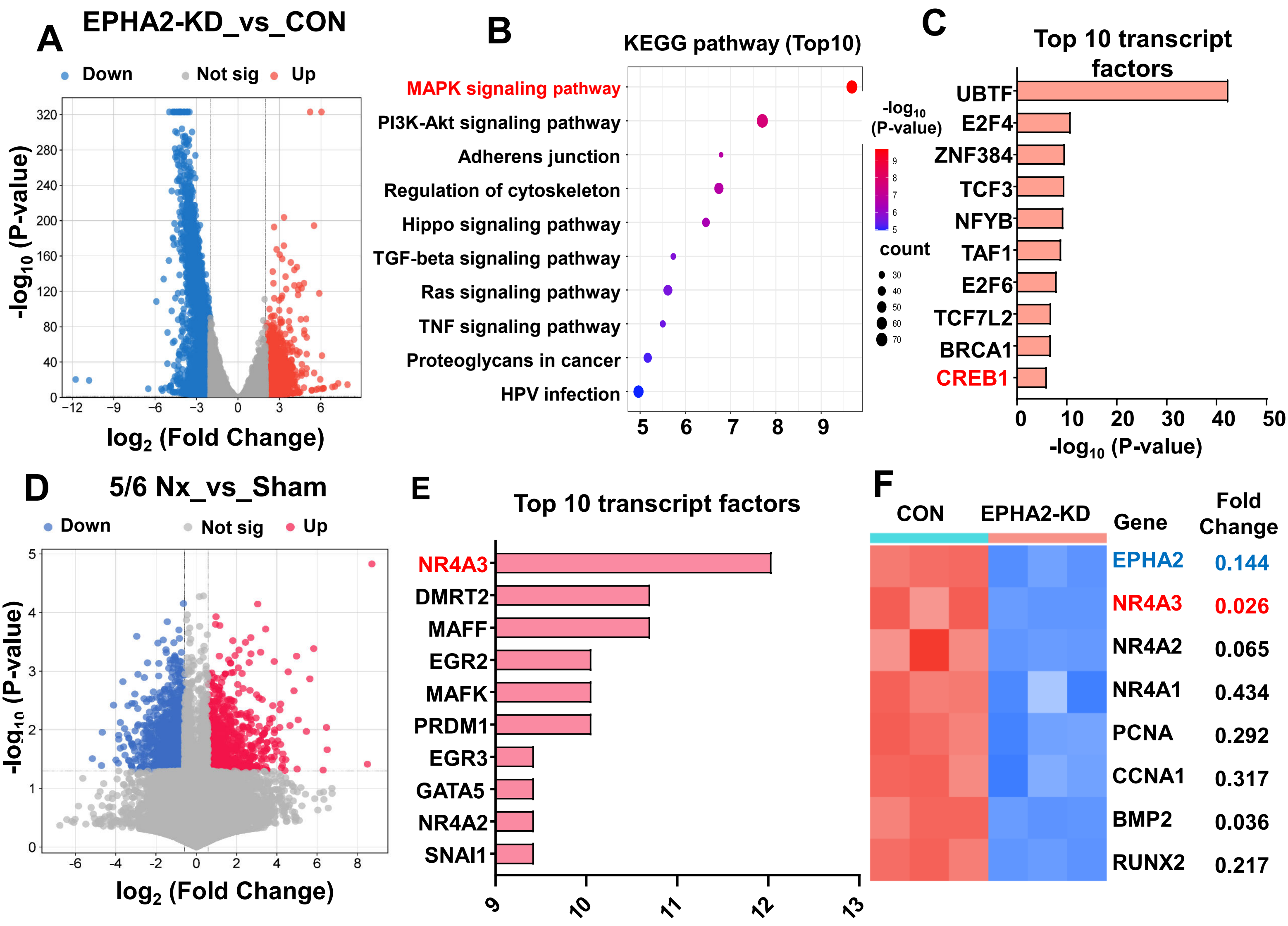

Figure S9. Transcriptomics Suggests EphA2 Regulates Vascular Stiffness via CREB and NR4A3.

(A) HASMCs were seeded on 20 kPa PA gels, and transfected with scrambled siRNA or siRNA targeting EPHA2. Volcano plot displaying differentially expressed genes (DEGs). Up- and down-regulated DEGs (*P*-adj<0.05, |log2FoldChange| > 2) are highlighted in red and blue, respectively. **(B)** KEGG pathway analysis of the 2762 down-regulated genes in EPHA2-KD vs. CON group. **(C)** Transcription factor (TF) enrichment analysis of the 2762 down-regulated genes in EPHA2-KD vs. CON group. **(D)** Volcano plot displaying differentially expressed genes (DEGs) in the aortas of sham or 5/6 nephrectomized mice. The upregulated genes (P value<0.05, fold change>1.5) and downregulated genes (P value<0.05, fold change<0.67) are highlighted in red and blue, respectively. **(E)** TF enrichment analysis of the 693 upregulated genes in 5/6 Nx vs. Sham group. **(F)** Heatmap showing the indicated genes in the control and EPHA2-KD group.

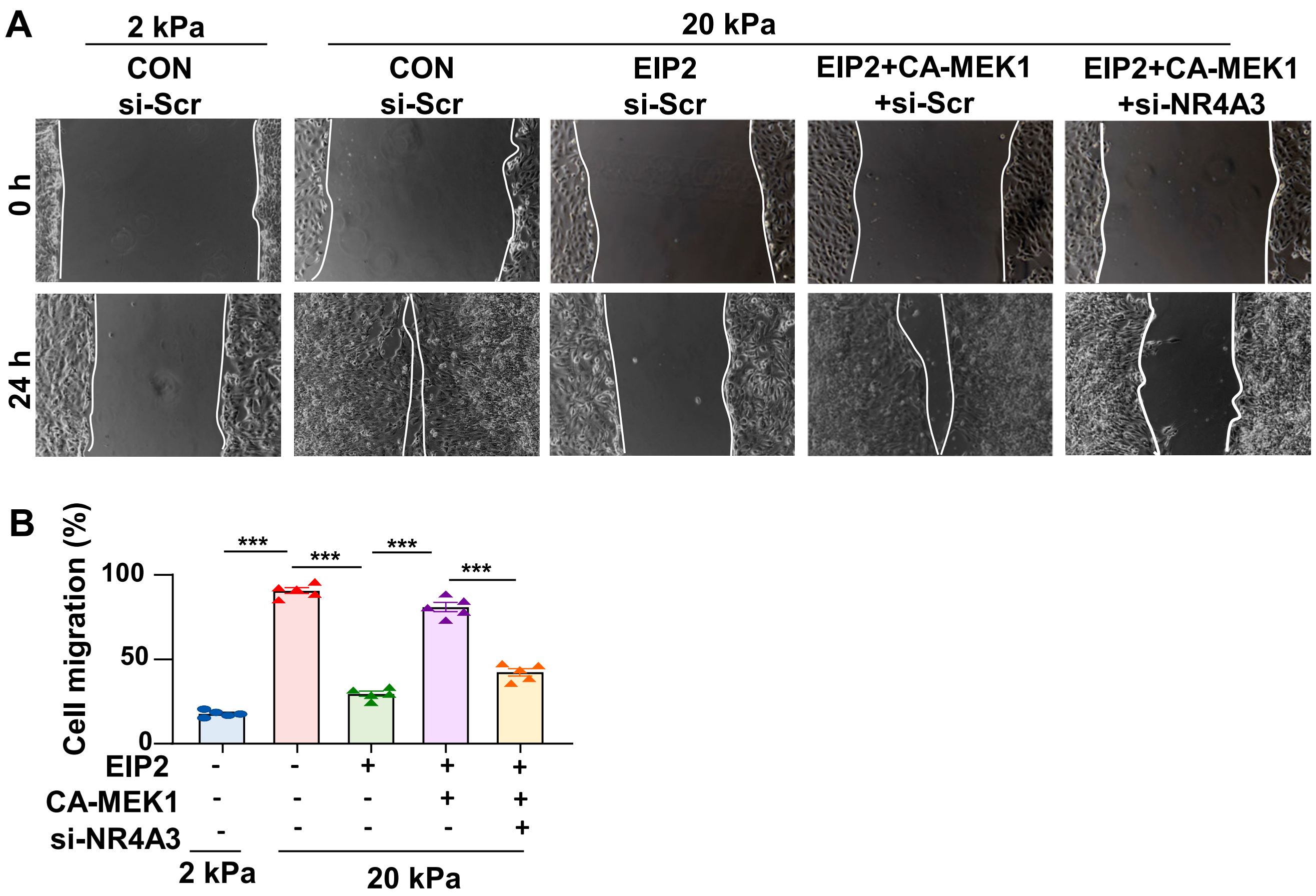

Figure S10. The EphA2-ERK-NR4A3 axis regulates stiffness-induced SMC migration.

(A) Representative images of a scratch wound healing assay performed with HASMCs on stiff gels. **(B)** Quantitative analysis of migration, expressed as percentage wound closure. n = 5 biological replicates. Data were expressed as the means ± SEM and analyzed by two-way ANOVA followed by Tukey's multiple comparison test.

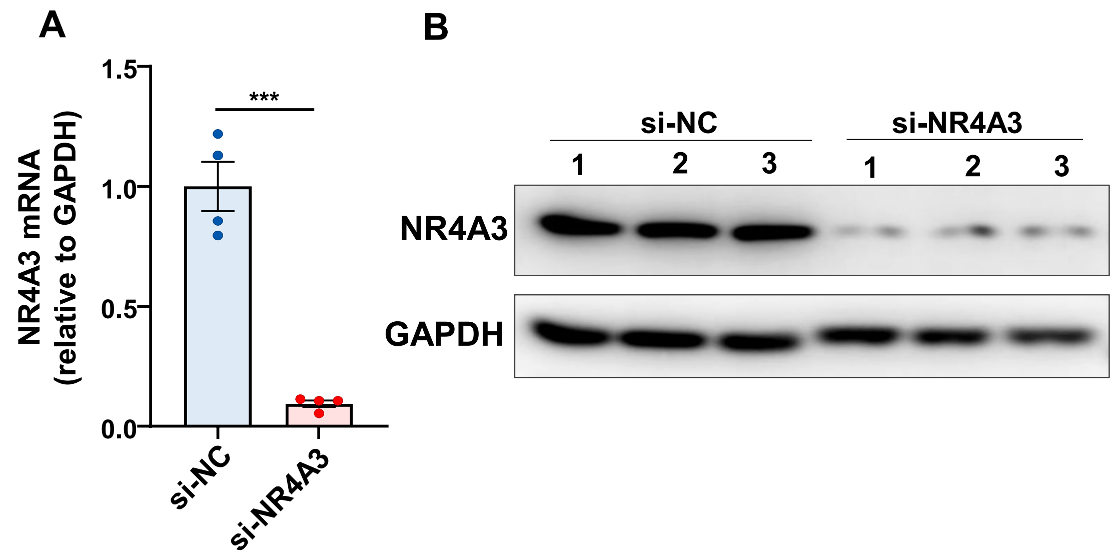

Figure S11. Verification of NR4A3 siRNA knockdown efficiency

(A) The knockdown efficiency of NR4A3 in HASMCs assessed by RT-qPCR. The cells were transfected with scrambled siRNA or NR4A3 targeted siRNA. RNA was extracted 24h after transfection. n = 4 biological replicates. Data were expressed as the means ± SEM and analyzed by Mann-Whitney test. **(B)** The knockdown efficiency of NR4A3 in VSMCs assessed by Western blot. The cells were transfected with scrambled siRNA or NR4A3 targeted siRNA. Protein was extracted 2 days after transfection. n = 3 biological replicates.

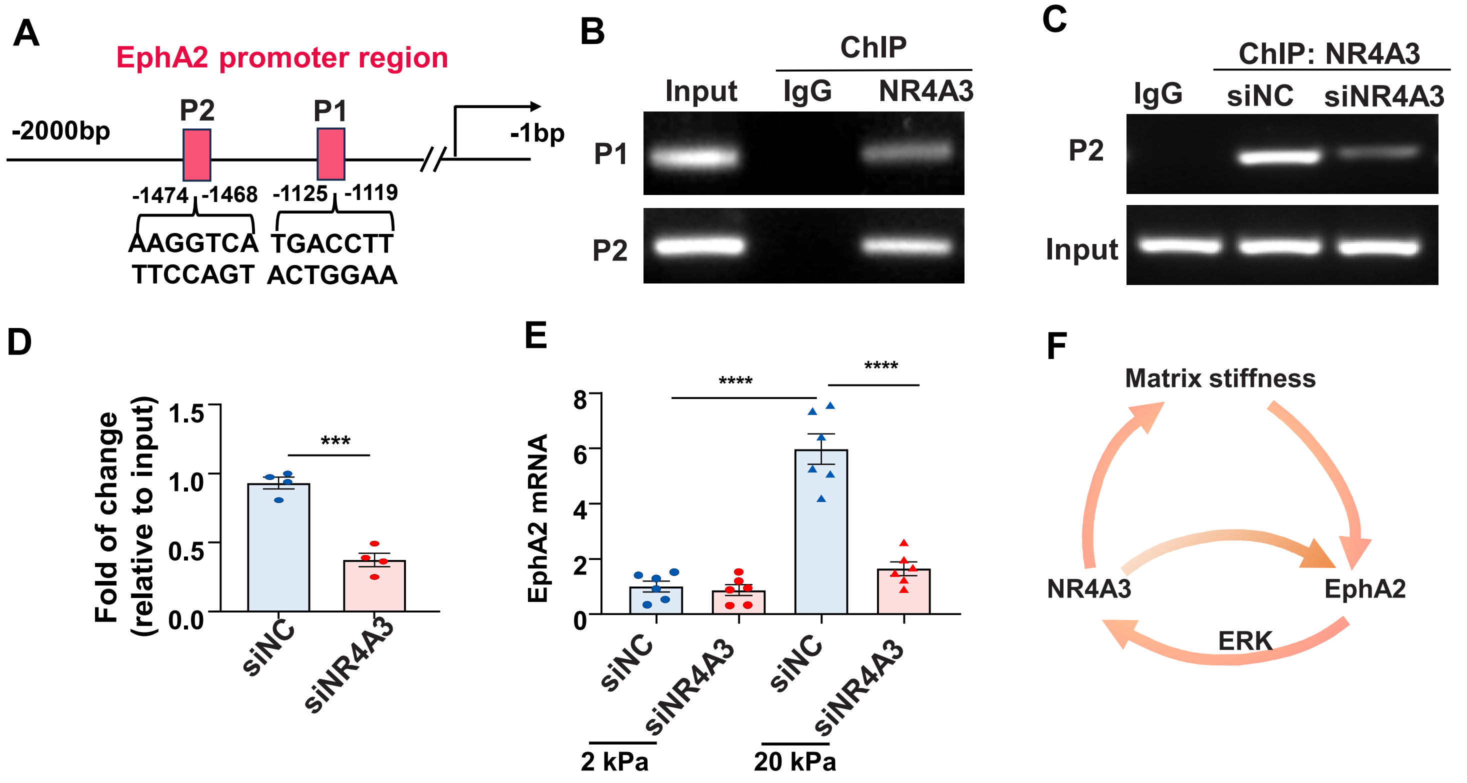

Figure S12. NR4A3 is responsible for stiffness-induced EphA2 expression and forms a positive feedback loop.

(A) Schematic illustration showing 2 sites in NR4A3 predicted to bind to the EphA2 promoter. **(B)** ChIP-PCR assay demonstrating the binding of NR4A3 to the EphA2 promoter. Chromatin from HASMCs seeded on 20 kPa gels was immunoprecipitated with an anti-NR4A3 antibody or control IgG. **(C)** ChIP-PCR assay performed in HASMCs transfected with NR4A3 siRNA or scrambled siRNA and seeded on 20 kPa gels for 48 hours. **(D)** quantification of the ChIP-PCR results. n = 4 biological replicates. The data was analyzed via unpaired t-test. **(E)** Quantitative RT-PCR analysis of the expression of EphA2 in HASMCs seeded on 2 kPa or 20 kPa gels and transfected with NR4A3 siRNA or scrambled siRNA. n = 6 biological replicates. The data were analyzed via two-way ANOVA followed by Tukey's multiple comparison test. **(F)** The schematic depicts an EphA2-ERK-NR4A3 positive feedback loop in mechanotransduction. The data were expressed as the means ± SEMs.

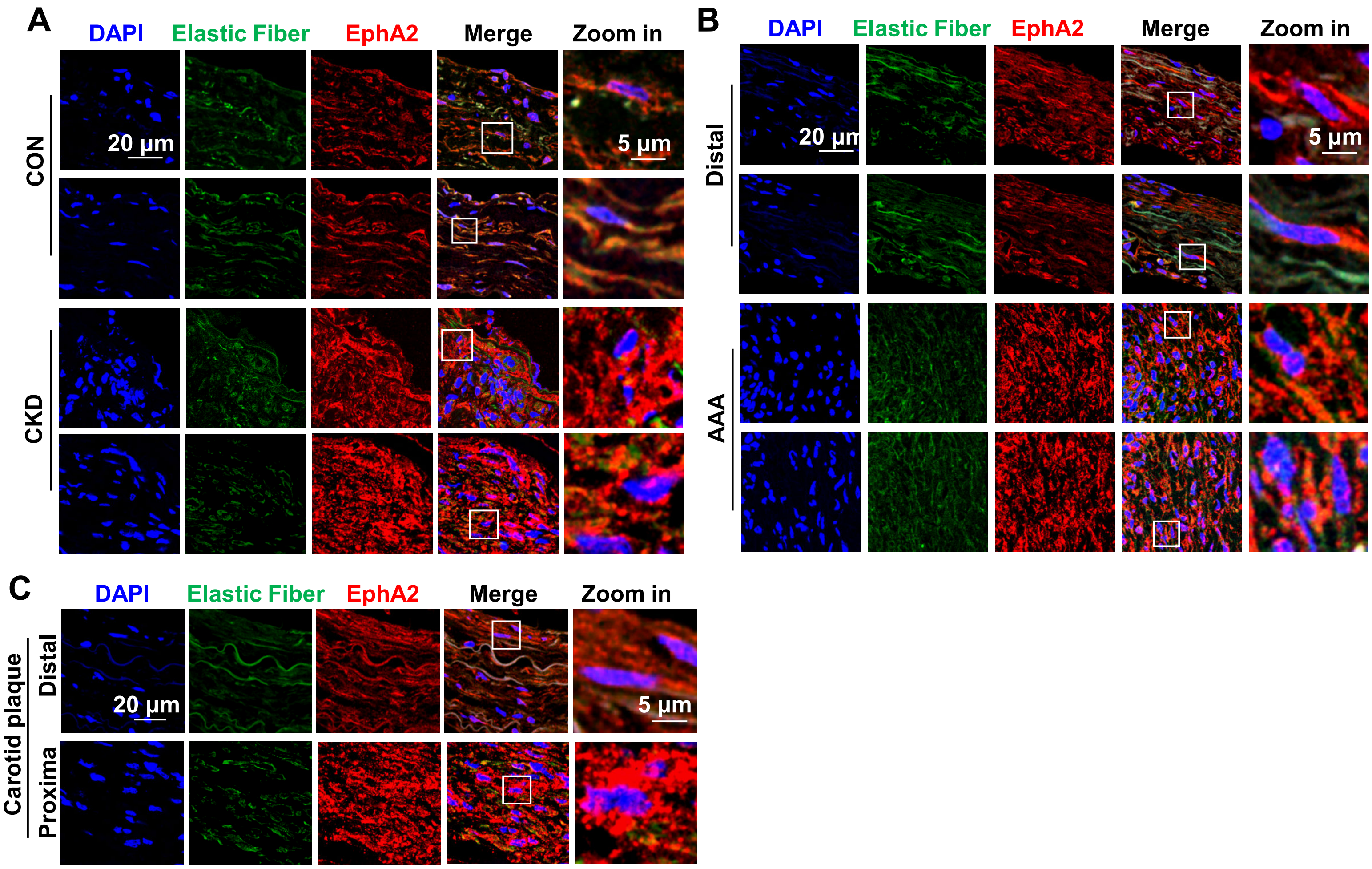

Figure S13. EphA2 exhibits increased liquid droplet formation in human diseased arteries. **(A–C)** Representative immunofluorescence images showing EphA2 condensates (red) in human artery sections from **(A)** popliteal arteries from chronic kidney disease (CKD) versus trauma-induced amputation (CON), **(B)** abdominal aortic aneurysm (AAA), and **(C)** carotid endarterectomy samples. Nuclei are stained with DAPI (blue). The green signal in the vessel wall represents autofluorescence of elastic fibers, which was natively excited by the 488-nm laser line.

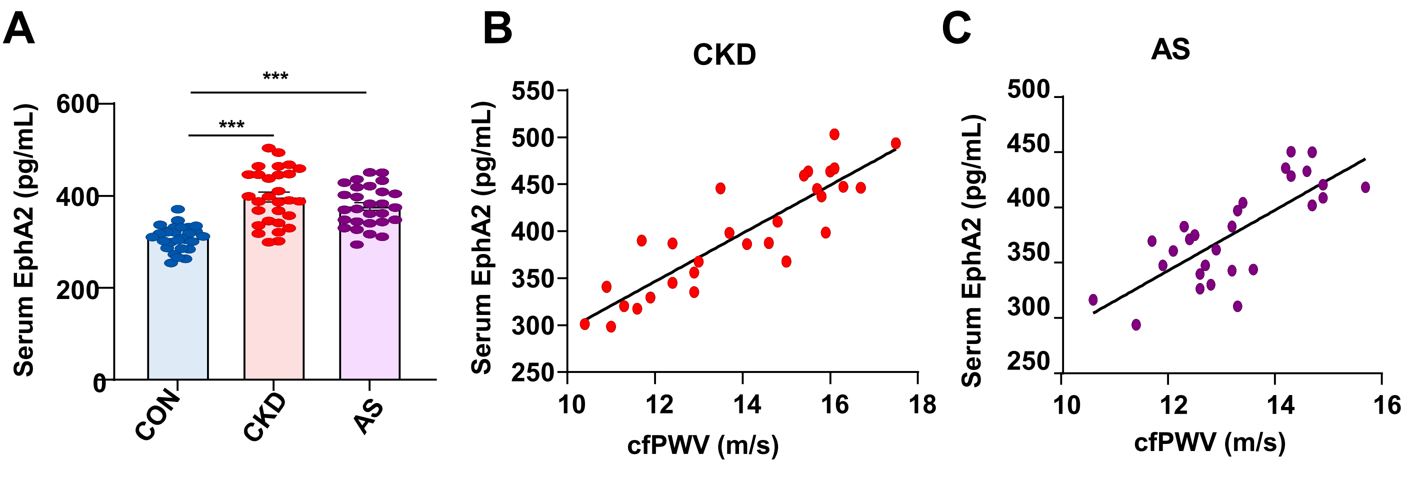

Figure S14. Analysis of serum EphA2 levels and their correlation with arterial stiffness in human.

(A) Bar graph comparing serum EphA2 concentrations across healthy control subjects, patients with chronic kidney disease (CKD), and patients with atherosclerosis. Data are presented as mean ± SEM, and compared using one-way ANOVA. The CKD cohort included patients with documented kidney damage or reduced glomerular filtration rate (eGFR < 60 mL/min/1.73 m^2) for >3 months, excluding those with recent (3-month) acute cardiovascular events or other major systemic inflammatory conditions. The Atherosclerosis cohort comprised patients with established atherosclerotic cardiovascular disease (e.g., coronary stenosis ≥50%, prior ischemic stroke with arterial stenosis, or symptomatic peripheral artery disease), while strictly excluding individuals with concurrent CKD (eGFR < 60mL/min/1.73m^2 or albuminuria) to ensure etiological distinction from the CKD cohort. **(B-C)** Scatter plots with linear regression lines demonstrating the correlation between serum EphA2 levels and carotid-to-femoral pulse wave velocity (cf-PWV), a measure of arterial stiffness, within CKD patients **(B)**, and patients with atherosclerosis **(C)**.

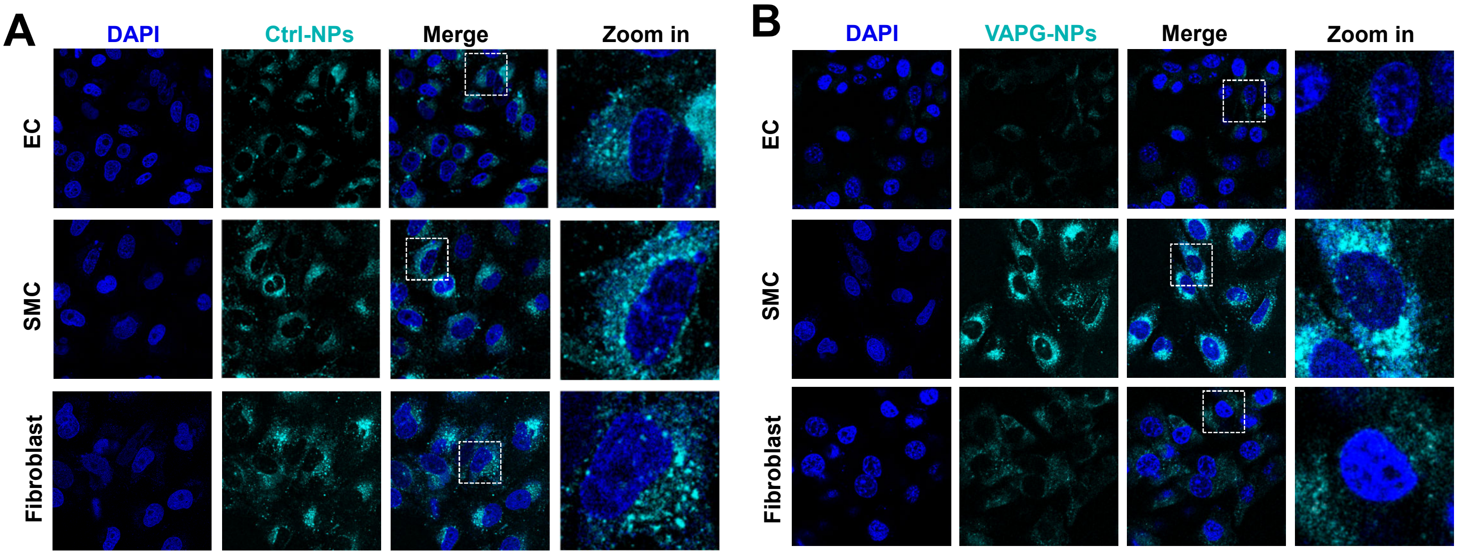

Figure S15. Uptake efficiency of Ctrl-NPs and VAPG-NPs in different vascular cells. **(A)** and **(B)** Representative fluorescence microscopy images showing the uptake of unmodified Ctrl-NPs or VAPG-NPs by endothelial cell (EC), smooth muscle cell (SMC), and adventitial fibroblasts. The prolonged culture period allowed for the accumulation of endogenous ECM proteins. NPs were incubated at a concentration of 20 μg/mL for 4 hours.

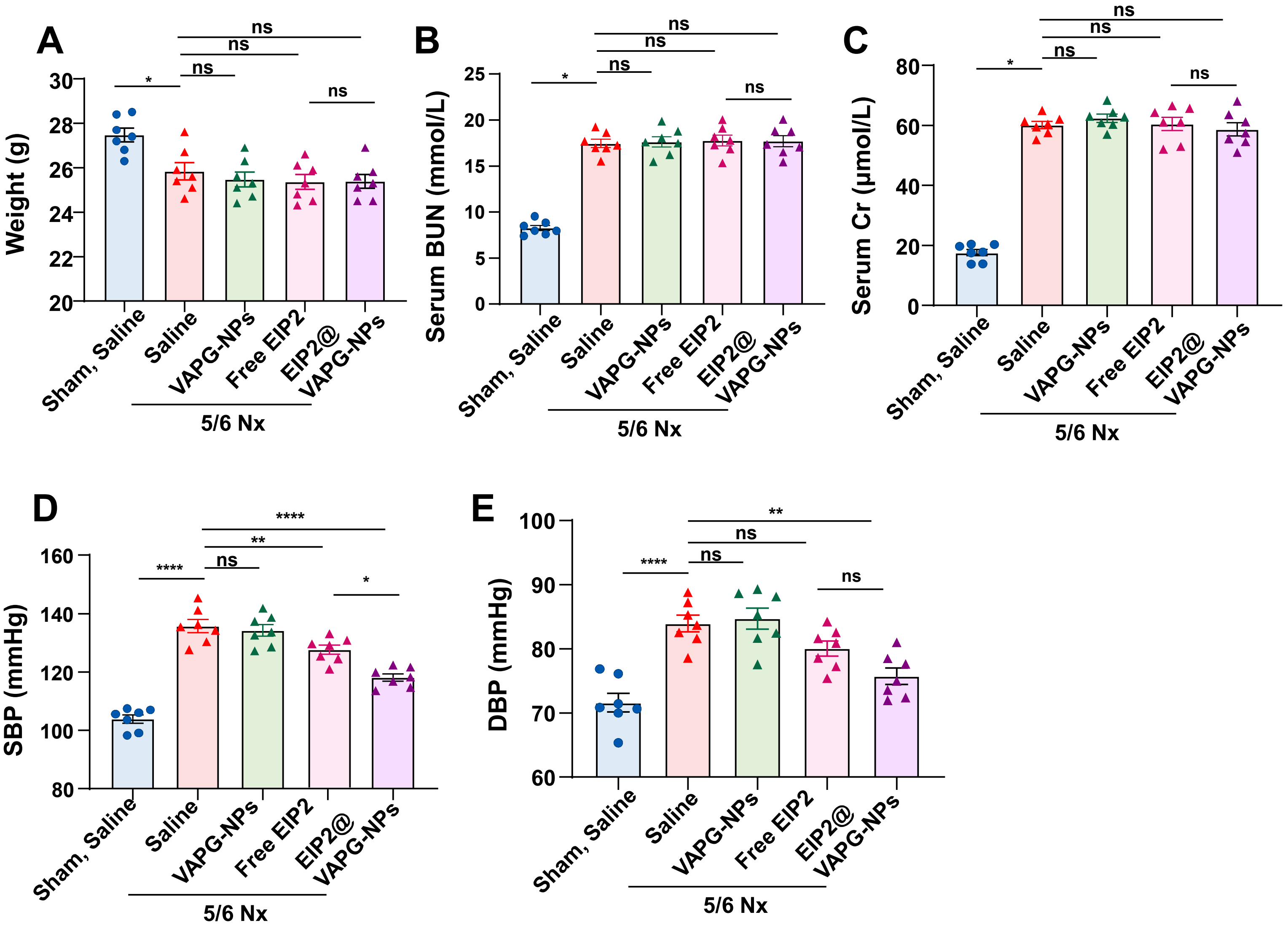

Figure S16. Physiological and biochemical indicators of the C57BL/6J mice subjected to 5/6 Nx or sham operation, with empty VAPG-NPs, free EIP2, or EIP2@VAPG-NPs administration.

(A) Body weight of mice from the indicated groups. **(B)** Serum level of blood urea nitrogen and **(C)** creatinine of mice from the indicated groups. n = 7 mice. Data were expressed as the means ± SEM and analyzed by two-way ANOVA followed by the Tukey's multiple Comparison. BUN, blood urea nitrogen; Cr, creatinine. **(D)** and **(E)** Systolic blood pressure (SBP) and diastolic blood pressure (DBP) of mice from the indicated groups. n = 7 mice.

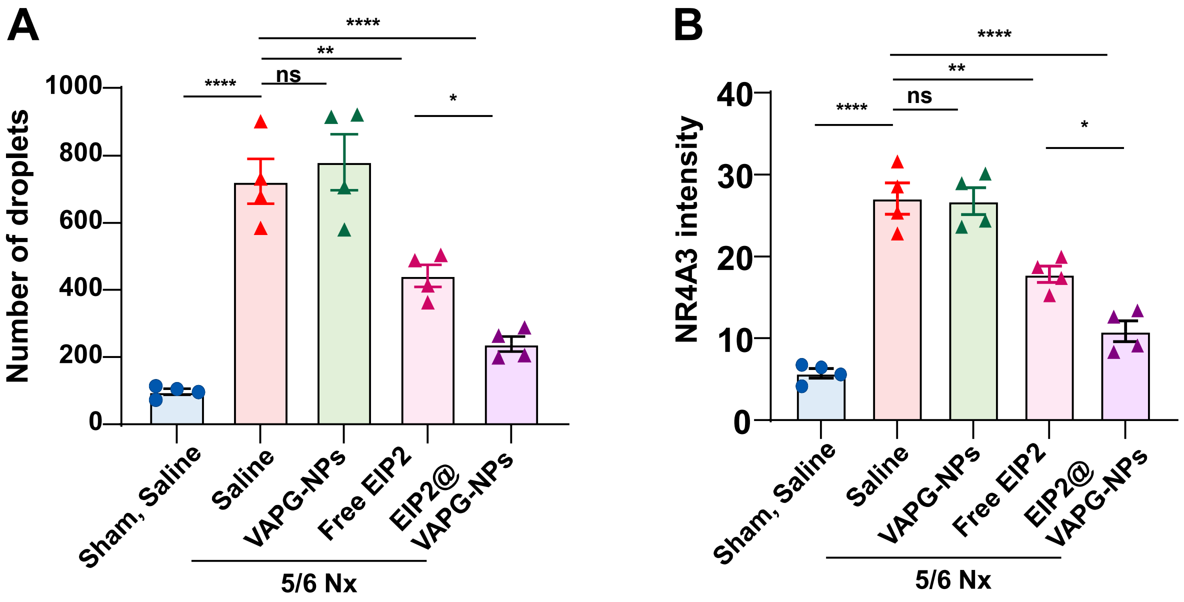

Figure S17. EphA2 droplets number and NR4A3 intensity in aorta.

(A) Quantification of EphA2 droplets number in control and 5/6 Nx mice, which were subjected to intraperitoneal injection of empty VAPG-NPs, free EIP2, or EIP2@VAPG-NPs every three days. n=4 mice. (B) Quantification of NR4A3 intensity in the indicated mice. n = 4 mice. The data were expressed as the means ± SEMs and analyzed via two-way ANOVA followed by Tukey's multiple comparison test.
