# Supplementary material for: Targeting mechanosensitive EphA2 phase separation to alleviate arterial stiffening
Source: Bioact Mater. 2026 Jan 24;60:203–15. doi: 10.1016/j.bioactmat.2026.01.020 (PMC12860789; doi:10.1016/j.bioactmat.2026.01.020)
Supplement: Multimedia component 2 [file mmc2.docx]

**SUPPLEMENTARY TABLES**

**Table S1: The baseline characteristics of control, CKD patients, and atherosclerosis patients**

| Characteristic | Control Group (n=28) | CKD Group (n=29) | *P1* value | AS Group (n=29) | *P2* value |
| --- | --- | --- | --- | --- | --- |
| Age (years) | 57.7 ± 9.1 | 61.0 ± 6.7 | 0.26 | 59.2 ± 8.0 | 0.27 |
| Sex (Male) | 15 (53.6%) | 16 (55.2%) | 0.903 | 15 (51.7%) | 0.889 |
| BMI (kg/m^2^) | 24.7 ± 2.9 | 26.0 ± 3.4 | 0.27 | 25.9 ± 3.3 | 0.34 |

P1 value: Compares the CKD group with the Control group. P2 value: Compares the AS group with the Control group. (one-way ANOVA for Age and BMI; Chi-square test for Sex)

**Table S2: Primers for genotyping**

| Primer | sequence(5'to3') |
| --- | --- |
| F-Epha2-flox | ACTTCTCCCAGGGTTAAGATGATT |
| R-Epha2-flox | AAGAAAAGGCTGTTGACAAGAGGG |
| F-SMMHC-Cre | TGACCCCATCTCTTCACTCC |
| R-SMMHC-Cre | AGTCCCTCACATCCTCAGGTT |

**Table S3: Primers for qPCR**

| Primer | sequence(5'to3') |
| --- | --- |
| F-EPHA2 | TGGCTCACACACCCGTATG |
| R-EPHA2 | GTCGCCAGACATCACGTTG |
| F-NR4A3 | CATACAGCTCGGAATACACCAC |
| R-NR4A3 | CCCTCCACGAAGGTACTGATG |
| F-MYH11 | CGCCAAGAGACTCGTCTGG |
| R-MYH11 | TCTTTCCCAACCGTGACCTTC |
| F-ACTA2 | CTATGAGGGCTATGCCTTGCC |
| R-ACTA2 | GCTCAGCAGTAGTAACGAAGGA |
| F-PCNA | GCAGAGTGGTCGTTGTCTTT |
| R-PCNA | TTGAGTGCCTCCAACACCTT |
| F-CCNA1 | GAGGTCCCGATGCTTGTCAG |
| R-CCNA1 | GTTAGCAGCCCTAGCACTGTC |
| F-BMP2 | TTCGGCCTGAAACAGAGACC |
| R-BMP2 | CCTGAGTGCCTGCGATACAG |
| F-RUNX2 | TGGTTACTGTCATGGCGGGTA |
| R-RUNX2 | TCTCAGATCGTTGAACCTTGCTA |
| F-GAPDH | AAGGTGAAGGTCGGAGTCAA |
| R-GAPDH | AATGAAGGGGTCATTGATGG |
| F-m-Epha2 | GCACAGGGAAAGGAAGTTGTT |
| R-m-Epha2 | CATGTAGATAGGCATGTCGTCC |
| F-m-Gapdh | AGGTCGGTGTGAACGGATTTG |
| R-m-Gapdh | TGTAGACCATGTAGTTGAGGTCA |

**Table S4: Primers for plasmid construction**

| Plasmid | Primer | sequence(5'to3') |
| --- | --- | --- |
| pcDNA-EGFP-EPHA2 | F-EGFP | AGCTTGGTACCGAGCTCATGGTGAGCAAGGGCGAG |
|  | R-EGFP | ACCAGAGCCCTTGTACAGCTCGTCCATGCC |
|  | F-EPHA2 | GCTGTACAAGGGCTCTGGTATGGAGCTCCAGGCAGC |
|  | R-EPHA2 | GTGCTGGATATCTGCATTAGATGGGGATCCCCACAGTG |
| pcDNA-EGFP-EPHA2-ΔLBD | F-EGFP | AGCTTGGTACCGAGCTCATGGTGAGCAAGGGCGAG |
|  | R-EGFP | ACCAGAGCCCTTGTACAGCTCGTCCATGCC |
|  | F-EphA2-ΔLBD | GCTGTACAAGGGCTCTGGTTATAAAAAGTGTCCACTCACAGTCCG |
|  | R-EphA2-ΔLBD | GTGCTGGATATCTGCATTAGATGGGGATCCCCACAGTG |
| Opto-FL | F-CRY2 | CTTGGTACCGAGCTCATGAAGATGGACAAAAAGAC |
|  | R-CRY2 | AGCTCCATACCAGAGCCTCCGGATCC |
|  | F-EPHA2-FL | GGAGGCTCTGGTATGGAGCTCCAGGCAGC |
|  | R-EPHA2-FL | GTGCTGGATATCTGCATTAGATGGGGATCCCCACAGTG |
| Opto-N1 | F-CRY2 | CTTGGTACCGAGCTCATGAAGATGGACAAAAAGAC |
|  | R-CRY2 | AGCTCCATACCAGAGCCTCCGGATCC |
|  | F-EPHA2-N1 | GGAGGCTCTGGTATGGAGCTCCAGGCAGC |
|  | R-EPHA2-N1 | GTGCTGGATATCTGCATTAATGGATCTCGGTAGTGAACTTCAAC |
| Opto-N2 | F-CRY2 | CTTGGTACCGAGCTCATGAAGATGGACAAAAAGAC |
|  | R-CRY2 | AGCTCCATACCAGAGCCTCCGGATCC |
|  | F-EPHA2-N2 | GGAGGCTCTGGTATGGAGCTCCAGGCAGC |
|  | R-EPHA2-N2 | GTGCTGGATATCTGCATTACACCGCCAAGTTGCCAG |
| Opto-C1 | F-CRY2 | CTTGGTACCGAGCTCATGAAGATGGACAAAAAGAC |
|  | R-CRY2 | CCGCCAATACCAGAGCCTCCGGATCC |
|  | F-EPHA2-C1 | GGAGGCTCTGGTATTGGCGGCGTGGCT |
|  | R-EPHA2-C1 | GTGCTGGATATCTGCATTAGATGGGGATCCCCACAGTG |
| Opto-C2 | F-CRY2 | CTTGGTACCGAGCTCATGAAGATGGACAAAAAGAC |
|  | R-CRY2 | CAGGATGGACCAGAGCCTCCGGATCC |
|  | F-EPHA2-C2 | GGAGGCTCTGGTCCATCCTGTGTCACTCGGC |
|  | R-EPHA2-C2 | GTGCTGGATATCTGCATTAGATGGGGATCCCCACAGTG |

**Table S5: Sequence for siRNA**

| siRNA name | Target sequence (5'to3') |
| --- | --- |
| siEPHA2 | CCATCAAGATGCAGCAGTATA |
| siNR4A3 | GCAGACATACAGCTCGGAATA |

**Table S6: Primers for ChIP-PCR**

| Primer | Sequence (5'to3') |
| --- | --- |
| F-Promoter1 | GCAACTGGGTTTGTTCAAGGCC |
| R-Promoter1 | TGTCTCTGAGCCTCCATCTCCTC |
| F-Promoter2 | GGGATTTCACCATGTTGGCCA |
| R-Promoter2 | TAGAACTCTGGTGCTGGGCG |

**Table S7: Nanoparticle size and zeta potential**

|  | EIP2@Ctrl-NPs | EIP2@VAPG-NPs | Empty@VAPG-NPs |
| --- | --- | --- | --- |
| Size (nm) | 133.12 ± 8.49 | 137.39 ± 10.38 | 117.86 ± 8.32 |
| Zeta potential (mV) | -3.04 ± 0.34 | -2.94 ± 0.33 | -15.26 ± 0.77 |

**Table S8: Drug encapsulation efficiency of drug-loading nanoparticles**

|  | EIP2@Ctrl-NPs | EIP2@VAPG-NPs |
| --- | --- | --- |
| Carrier concentrations (mg/mL) | 10.00 mg/mL | 10.00 mg/mL |
| Drug concentration (mg/mL) | 0.98 mg/mL | 0.91 mg/mL |
| Coagulant dosage (mg) | 7.00 mg | 7.00 mg |
| Drug loading rate (%) | 8.92% | 9.10% |
| Entrapment rate (%) | 70% | 65% |
