# Supplementary material for: Targeting mechanosensitive EphA2 phase separation to alleviate arterial stiffening
Source: Bioact Mater. 2026 Jan 24;60:203–15. doi: 10.1016/j.bioactmat.2026.01.020 (PMC12860789; doi:10.1016/j.bioactmat.2026.01.020)
Supplement: Multimedia component 3 [file mmc3.docx]

**Expanded Methods**

**Cell culture**

Human aortic smooth muscle cells (HASMCs) were obtained from Procell (CP-H081). Human umbilical vein endothelial cells (HUVECs) were obtained from Procell (CP-H082). Human vascular adventitial fibroblasts (HVAFs) were obtained from Procell (CP-H200). All cells were authenticated using short tandem repeats (STR) testing. HASMCs were cultured in commercial complete medium (CM-H081; Procell). HUVECs were cultured in commercial complete medium (CM-H082; Procell). HVAFs were cultured in commercial complete medium (CM-H200; Procell). Collagen I (#354236; Biocoat) at 50 µg/ml was coated on polyacrylamide gel surfaces.

**Antibodies**

Primary antibodies against EphA2 (#66736-1-Ig; Proteintech), Phospho-EphA2-S897 (AP1082; Abclonal), CREB (#9197; CST), Phospho-CREB (#9198, CST), ERK1/2 (#11257-1-AP; Proteintech), Phospho-ERK1/2 (#28733-1-AP; Proteintech), NR4A3 (#55405-1-AP; Proteintech), RAF1 (#66592-1-Ig; Proteintech), MEK1/2 (#11049-1-AP; Proteintech) and β-Tubulin (#10094-1-AP; Proteintech) were used for Western blotting and immunofluorescence staining.

**Polyacrylamide (PA) gel preparation**

PA gels with different stiffness were obtained by varying the final concentrations of acrylamide (A4058; Sigma-Aldrich) and bis-acrylamide (M1533; Sigma) for the corresponding elastic moduli. The formula of 2 kPa hydrogel solution is 850 μl deionized water, 50 μl 2% methylenebisacrylamide solution and 100 μl 40% acrylamide solution; and the formula of 20 kPa hydrogel solution is 668 μl deionized water, 132 μl 2% methylenebisacrylamide solution, 200 μl 40% acrylamide solution. The mixtures were degassed for 20 minutes to remove oxygen from the solutions. To polymerize the mixtures, 6 μl of 10% w/v ammonium persulfate (Bio-Rad) and 3 μl of TEMED were added into the above mixed hydrogel solution.To cross-link extracellular matrix proteins onto the gel surface, the gels were activated by exposing the heterobifunctional crosslinker sulfo-SANPAH (Pierce, 0.5 mg/ml in PBS) to UV 365 nm light for 10 min and subsequently were coated with 400 ml of Collagen I (50 µg/ml, Corning) for each gel at 37°C for 1 hour.

**In situ stiffening bioclick hydrogels**

The hydrogels were formed via a copper-free click reaction (SPAAC) between azides and activated cyclooctynes, which can further undergo an in situ stiffening through a secondary photopolymerization of unreacted alkyne species to model ECM stiffening ^23^. 4ARM PEG-DBCO (20,000 g/mol) and 4ARM PEG-N3 (5000 g/mol) and N3-GRGDS were purchased from Xi'an Qiyue Biology. PEG-DBCO (5%, w/v) and N3-KRGDS (1 mM) were pre reacted on ice for 5 min. Then, PEG-N3 was added at different concentrations to obtain DBCO: N3 ratio of 4:1. To obtain in situ stiffening hydrogels, 2 mM lithium phenyl-2,4,6 trimethylbenzoylphosphinate (LAP) was also added to the hydrogel solution. The mixture was vortexed, and the 15 μl of the gel solutions were sandwiched between a sigma-coated (Sigma-Aldrich) glass cover slide and an azide-functionalized 12 mm × 12 mm glass coverslip, In the presence of photoinitiator (LAP), the network formed by excessive DBCO can be further strengthened under 365 nm light irradiation, and the elastic modulus of the hydrogel can be increased from 2 kPa to 32 kPa within 120s.

**Animals and the 5/6 nephrectomy surgery**

All procedures were approved by the Animal Care and Use Committee of Fujian Medical University and approved by the Ethics Committee of Fujian Medical University (IACUC FJMU 2025-0224). Wildtype mice (C57BL6J, 8-10 weeks old, male) were obtained from the Experimental Animal Center at Fujian Medical University (Fuzhou, China) and housed in a temperature-controlled room with 12-hour light-dark cycles with free access to food and water. For 5/6 nephrectomy, mice were received a preoperative subcutaneous injection of carprofen (5 mg/kg) for long-lasting analgesia. Anesthesia will be induced in an induction chamber using 3.0% - 4.0% isoflurane delivered in oxygen. Following the loss of righting reflex, the animal will be transferred to a nose cone for maintenance on a surgical plane of anesthesia with 1.5% - 2.0% isoflurane. The depth of anesthesia will be continuously monitored and the isoflurane concentration adjusted accordingly in response to vital signs and response to noxious stimuli. The left kidney was subjected to 2/3 nephrectomy conducted by amputation of the superior and inferior renal poles. Right kidney nephrectomy was carried out one week later. Sham surgery was also performed as the two-step procedure in which the respective kidney was exposed and then repositioned. Mice were fed on a diet containing 2% phosphate for 7 weeks after 5/6 nephrectomy. The success criterion for modeling is that after 7 weeks, the detection of mice blood creatinine, urea nitrogen indicates successful induction of renal failure.

**VSMC-specific knock out of Epha2 in mice**

Epha2-flox mice (Strain S-CKO-18324) were purchased from Cyagen. Mice with transgene CreER^T2^ under the control of SMC-specific myosin heavy chain promoter (Myh11-Cre) was obtained from the Jackson Laboratories (stock number 019079). In the Myh11-CreER^T2^ mice, the bacterial artificial chromosomes (BAC) transgene was inserted into the Y Chromosome, thus only male mice are able to express the Cre recombinase. Epha2^flox/flox^ mice were maintained in a C57BL/6J background and crossed with Myh11-CreER^T2^ mice, to generate the Epha2^flox/flox^ Myh11-CreER^T2+^ (Epha2^sKO^) mice. Epha2^flox/flox^ Myh11-CreER^T2-^ (Epha2^WT^) littermates were used as the controls. Primers for genotyping are listed in Table S2. Mice were subjected to tamoxifen (dissolved in corn oil) intraperitoneal injection with 40 mg/kg every day for 7 days.

**Tail-cuff measurement of blood pressure**

Blood pressure was recorded by using a CODA Mouse & Rat Tail-Cuff Blood Pressure System (Kent Scientific Co., Connecticut, USA). Mice were placed in the restraint corridor and allowed at least 10 minutes of acclimation. The area was warmed with a heating pad and a quiet, dark environment was maintained to ensure reliable measurements within the parameters of this technology. The mice underwent 3 consecutive days of training sessions from 3 to 6 PM each day to become accustomed to the tailcuff procedure. 15 cycles per measurement were daily performed on each mouse and the blood pressure was the mean value of all successful measurements.

**Vascular stiffness measured by ultrasound**

Vascular stiffness was assessed by pulse wave velocity (PWV) using a Vevo LAZR system (FUJIFILM VisualSonics, Canada). Mice were anaesthetized by 1.5-2% isoflurane and placed on the platform that carries electrocardiogram (ECG) electrodes. MX transducer MX550D was used. The vascular pulse-wave Doppler tracing was recorded at the position of carotid bifurcation or proximal aortic arch (position 1). The time from the onset of the QRS to the onset of the Doppler waveform was measured (T1). Then another position in the distal area from the carotid bifurcation or proximal descending aorta was selected and the pulse-wave Doppler tracing was recorded (position 2). The time from the onset of the QRS to the onset of the Doppler waveform was measured (T2). The distance between position1 and position2 was measured (L). PWV is calculated as: L/(T1–T2) (m/s).

**Measurement of vascular stiffness with nanoindentation**

The elastic moduli of samples were measured by nanoindentation as previously described, the pulmonary arteries of rats were cut longitudinally and placed on a glass slide with intima facing down after the adventitia was removed. During indentation, the tip was brought into contact with the tissue surface with a microscope to ensure accurate measurement. Probe with spring constants of 0.54 N/m and the spherical tips with the radius of 48 μm was used. Based on the load-displacement curves, the reduced Young’s modulus (RedYM) was calculated using the Hertz spherical indentation model.

**Collection of Human Vascular Tissue and Plasma Samples**

Human vascular tissue samples were collected from three clinical cohorts for immunofluorescence analysis: (1) popliteal arteries from patients with chronic kidney disease (CKD) and from control subjects undergoing trauma-induced amputation; (2) abdominal aortic aneurysm (AAA) specimens, with adjacent, less affected vessel regions serving as internal controls; and (3) carotid endarterectomy samples from patients with atherosclerosis, similarly using distal regions as internal controls. Corresponding peripheral blood plasma samples were also collected from a subset of these cohorts, comprising control subjects, patients with CKD, and patients with atherosclerosis. The CKD cohort included patients with documented kidney damage or reduced glomerular filtration rate (eGFR < 60 mL/min/1.73 m²) for >3 months, excluding those with recent (3-month) acute cardiovascular events or other major systemic inflammatory conditions. The Atherosclerosis cohort comprised patients with established atherosclerotic cardiovascular disease (e.g., coronary stenosis ≥50%, prior ischemic stroke with arterial stenosis, or symptomatic peripheral artery disease), while strictly excluding individuals with concurrent CKD (eGFR < 60 mL/min/1.73 m² or albuminuria) to ensure etiological distinction from the CKD cohort. Patients with recent acute cardiovascular events or other major confounding systemic diseases were also excluded. All procedures involving human samples were approved by the Medical Ethics Committee of the First Affiliated Hospital of Fujian Medical University (Approval Nos. [2025]216 and [2023]508) and were performed in accordance with ethical standards. Informed consent was obtained from all participants. Plasma EphA2 levels were quantified using a commercial Human EphA2 ELISA kit (EH173RB, Invitrogen), following the manufacturer's protocol. Arterial stiffness was clinically assessed by measuring carotid-femoral pulse wave velocity (cf-PWV).

**Alizarin Red S staining and Masson staining**

For Alizarin Red S staining, frozen sections were exposed to 0.2% Alizarin Red S (G1452; Solarbio) 30 min at 37°C followed by washing with 0.2% acetic acid. For Masson staining, frozen sections were stained with Masson's Trichrome Stain Kit (G1340; Solarbio).

**Calcification quantification**

The lysis buffer for aortic tissue and VSMCs was prepared in accordance with the instructions provided in the calcium colorimetric assay kit (Beyotime, China). Protein concentrations in the aortic tissue and VSMCs lysates were quantified using a BCA protein assay kit (Thermo Fisher Scientific; Cat. No. 23225). To assess calcium accumulation, the lysates of aortic tissue and VSMCs were analyzed colorimetrically following the manufacturer’s protocol. The calcium content in the lysates was normalized to the total protein content and expressed as micrograms of calcium per milligram of protein (µg Ca/mg protein).

**RNA isolation and quantitative RT-PCR**

Mouse aortic arteries were dissected and the endothelia and adventitia were removed by cotton swabs and microforceps, respectively. After that, the remaining tissues were crushed in porcelain mortar with 1 ml of TRIzol reagent (#15596026; Thermo Fisher) exposed to liquid nitrogen. RNA from cultured cells was also extracted by TRIzol reagent according to the manufacturer's instructions. Isolated RNAs were reversed-transcribed into complementary DNA by Transcriptor First Strand cDNA Synthesis Kit (Roche, Basel, Switzerland). qRT-PCR experiments were performed using the One Step TB Green® PrimeScript™ RT-PCR Kit (Takara, Japan) and the Multicolor Real-Time PCR Detection System (ABI, USA). All specific primers used for detection are listed in Table S3.

**Western blotting**

Cells were lysed in RIPA buffer (MP015; Macgene) containing protease inhibitor PMSF cocktail (CM00738; Abcam) and phosphatase inhibitors (#524628; Millipore). Protein concentration was determined with the Bicinchoninic Acid (BCA) assay by Pierce™ BCA Protein Assay Kit (23250; Thermo Fisher). An equal amount of protein samples (30-40 µg) was then subjected to 10% SDS-PAGE and electrophoretically transferred onto a polyvinylidene fluoride membrane (PVDF membrane). After being blocked with 5% (w/v) skim milk (Bio-rad) at room temperature for 1-2 hours, the membranes were incubated overnight at 4 ℃ with primary antibodies. Then, the PVDF membrane was incubated with horseradish peroxidase (HRP)-labeled second antibody (#7074 or #7076, Cell Signaling, 1:5000 dilution) at room temperature for 1-2 hours. Immunoreactive bands were detected with enhanced chemiluminescence (ECL) reagent (Thermo, Carlsbad, CA, USA) and the ChemiImager 5500V2.03 software.

**luciferase reporter assay**

The pGL4.29[luc2P/CRE/Hygro] Vector contains a cAMP response element (CRE) that drives the transcription of the luciferase reporter gene luc2P was purchased from Promega (#E8471). The 4×NR4A3 luciferase reporter was constructed using ClonExpress Ultra One Step Cloning Kit (C115-01; Vazyme) to ligate 4× clustered NR4A3 binding motif (AAAGGTCA) into the luciferase reporter (#10959; Addgene) opened by restriction digestion (BamHI and EcoRI). To detect the transcription activity of CRE or NR4A3, luciferase plasmid (or PGL-basic) and pSV-β-galactosidase plasmids were co-transfected into HASMCs. The initial number of cells plated in 60 mm dishes was approximately 2~3×10^6. For each 60 mm dish, 6 µl of lipofectamine 2000 transfection agent (#11668019; Thermo Fisher) was employed. At 24 hours post-transfection, cells were seeded on 2/20 kPa PA gels and cultured for 24 hours. Luciferase activity was measured using the luciferase assay system (RG005; Beyotime) and normalized to the β-galactosidase activity assessed using o-Nitrophenyl-β-D-galactopyranoside (#0789; Amresco).

**Plasmids and Transient transfection**

Plasmid containing full-length EPHA2 (human) was purchased from Miaoling Biology (P34713). Plasmid containing Cry2 (CRY2olig-mCherry) was purchased from Addgene (#60032) to generate the Opto-droplet optogenetic system. EGFP fragment was subcloned from pEGFP-N1(P0133; Miaoling Biology). All plasmids were constructed using ClonExpress Ultra One Step Cloning Kit (C115-01; Vazyme) to ligate PCR products into pcDNA3.1 backbone vector opened by restriction digestion (BamHI and EcoRI). DNA DNA sequence encoding MEK1-S218D/S222D is directly obtained through gene synthesis. All primers used for plasmid construction are listed in Table S4. All generated plasmid sequences were confirmed by DNA sequencing. Cell transfections were carried out by Lonza 4D-Nucleofector according to the manufacturer's instructions at room temperature. The reagents used for electroporation of HASMCs were P1 Primary Cell 4D-Nucleofector™ X Kit (V4XP-1012), and the electroporation programs were EH-106 (Lonza; Cat. No. CC-2571). 1×10^6 cells were transfected with 2 µg plasmid DNA. After electroporation according to the manufacturer’s instructions, the cells were cultured in a 37°C incubator for 24 h, and then seeded on 2/20 kPa PA gels. For knockdown of EPHA2 or NR4A3, cells at 80% confluence were transfected with siRNAs specific for EPHA2 or NR4A3 (Table S5).

**Immunofluorescence and live cell imaging**

For immunofluorescence, cells were fixed in 4% PFA for 10 minutes and permeabilized with 0.2% Triton X-100 in PBS for 5 minutes. Nonspecific binding was blocked by 5% BSA for 30 min at room temperature. The primary antibody was diluted 1:200 in PBS and incubated overnight at 4°C, then probed with secondary antibody including Alexa Fluor 555-conjugated goat anti-mouse IgG (ab150114; Abcam) or Alexa Fluor 488-conjugated goat anti-rabbit IgG (ab150077; Abcam) for 1 hour at room temperature. Nuclei were counterstained with DAPI. Images of either fixed cells or live cells were captured by using Leica SP8 confocal microscope.

**Fluorescence recovery after photobleaching (FRAP) assay**

Cells were transfected with EGFP-EphA2 at 48 hour before imaging. FRAP assay was performed by Leica SP8 confocal microscope. GFP signals in regions of interest (ROI) were bleached by using a 488-nm laser beam at 60% power. The fluorescence intensity between pre-bleaching and the time point right after bleaching was recorded by microscope. For FRAP curves fitting, logarithmic equation fitting was used.

**Recombinant proteins**

A DNA sequence encoding EphA2 (Uniprot No.:P29317, 538~976 aa) was cloned into pDG02583 (104129, Addgene) vector which opened by restriction digestion (EcoRI and HindIII) with an N-terminal His14-MBP-bdSUMO-EGFP-tag. The MBP-bdSUMO fusion tag significantly enhances the solubility of membrane proteins during expression. The large MBP domain acts as a solubility enhancer, while the bdSUMO tag allows for its precise and efficient removal following purification, yielding the native protein. The recombinant protein was expressed by Transetta (DE3) Chemically Competent Cell (CD801, TransGen Biotech), induced for 16 h by adding 0.2 mM IPTG at 16 ℃, purified by Ni-NTA column in buffer containing 20 mM Tris-HCl (pH 6.8), 500 mM NaCl, 1 mM DTT and 10% glycerin. Then the recombinant protein was cleaved by 300 nM bdSENP1 protease (#104962, Addgene) for 12 h to obtain EGFP-EphA2 recombinant protein. DNA sequence encoding ERK1 (Uniprot No.:P27361, 1~379aa) and ERK2 (Uniprot No.:P28482, 1~360aa) fused with N-terminal His6-mCherry tag was cloned into the pET32a vector. These above recombinant proteins were finally concentrated to 20 mM using Amicon Ultra centrifugal filter (Merck). For in vitro kinase activity assay, DNA sequence encoding EphA2 (Uniprot No.:P29317, 561~976 aa) and MEK1 (Uniprot No.:Q02750, 1~393 aa, S218D/S222D) fused with N-terminal GST tag were also cloned into the pET32a vector. These proteins were expressed by baculovirus in Sf9 insect cells to ensure their kinase activity, and purified by Ni-NTA column in buffer containing 50 mM Tris-HCl (pH=7.5), 300 mM NaCl, 10mM glutathione, 0.1 mM EDTA, 1.0 mM DTT and 10% glycerin.

**In vitro droplet assay**

Purified proteins were diluted to different concentrations with a buffer containing 20 mM Tris-HCl (pH 6.5), 75 mM KCl, and 1 mM DTT, and the mixtures were incubated at 4℃ for 5 minutes to induce phase separation. 10 µl of the turbid solution was injected into glass-bottom dish and imaged by Leica SP8 confocal microscope.

**Co-immunoprecipitation and identification of EphA2-binding proteins**

HASMCs were lysed with NETN buffer with 1× protease inhibitor cocktail (Roche) and 1× PMSF for 30 minutes on ice, followed by centrifugation at 12,000 rpm for 10 minutes at 4°C. Cell lysates of HASMCs cultured on 2/20 kPa were from 3 independent biological replicates, and each group containing 100 ug of proteome sample. 4 ul anti-EphA2 monoclonal antibody (Proteintech; 66736-1-Ig) was added to cell lysates and incubated overnight at 4°C on a ferris wheel. After that, 30 ul of protein A/G magnetic beads were added the tubes and incubated 1 hour at 4°C. Beads were subsequently washed three times with NETN buffer. Pull-down lysates were separated by SDS-PAGE and submitted for mass spectrometry analysis at oebiotech. Co-IP proteins were identified using an Orbitrap-Fusion mass spectrometer (Thermo Scientific) coupled with an Easy-nLC 1000 system (Thermo Scientific).

**TurboID proximity labeling**

Fuse TurboID to EphA2, express it in HASMCs, then seed cells on 2 kPa or 20 kPa PA gels, incubate cells with biotin for 15 min to label proximal proteins, then capture and identify biotinylated interactors via streptavidin pulldown and western blotting.

**Chromatin immunoprecipitation (ChIP)**

HASMCs were cultured on 20 kPa gels for 24 h. Then the cells were harvested and then washed twice with PBS, crosslinked with 1% formaldehyde in PBS for 10 min, rinsed twice with ice-cold PBS and resuspended with 300 µl of lysis bufer (1% SDS, 5 mM EDTA, 50 mM Tris-HCl (pH 8.1), and protease inhibitors), incubated on ice for 10 min, and sonicated for 3 times at 12 s each. 10% aliquot was saved as an input. The lysate was 1:10 diluted in dilution buffer (1% Triton X-100, 2 mM EDTA, 150 mM NaCl, 20 mM Tris-HCl (pH=8.1), and protease inhibitors), and was incubated with antibody against NR4A3 for 6 h or overnight at 4 °C and then with 30 μl of protein A-G sepharose beads for another 2 h. The sepharose beads were washed sequentially with buffer TSE I (0.1% SDS, 1% Triton X-100, 2 mM EDTA, 20 mM Tris-HCl (pH 8.1), 150 mM NaCl), buffer TSE II (0.1% SDS, 1% Triton X-100, 2 mM EDTA, 20 mM Tris-HCl (pH=8.1), 500 mM NaCl), buffer III (0.25 mol/L LiCl, 1% NP-40, 1% deoxycholate, 1 mM EDTA, 10 mM Tris-HCl (pH 8.1)), and TE buffer. Crosslinks were reversed at 65 °C overnight. DNA was extracted with DNA Pure-Spin Kit (Vigorous), and subjected for PCR amplification. Primers used for ChIP-PCR are listed in Table S6. Amplification was carried out as follows: 3 min at 95°C; 35 cycles of 30 s at 95°C, 30 s at 55°C, and 20 s at 72°C; and finally 10 min at 72°C. PCR products were loaded onto a 2% agarose gel, and the separated bands were documented using the Gel Doc XR+ System (BioRad).

**Interfering peptides**

The EIPs modified with d-retro-inverso (DRI) were as follows: EIP1, H-iettfklvaqnpdeythpdvytkPPRRRQRRKKRG-OH; EIP2, H-lpklqesksfyvdepsqrarqnkPPRRRQRRKKRG-OH; EIP3, H-rrrhiffgvgalvlllvvgvavggPPRRRQRRKKRG-OH. Scrambled1: H-tyvpkdteqyfhtlipkvdtaenpPPRRRQRRKKRG-OH; Scrambled2, H-sqkprlqeknqyfvsdrekalsqpPPRRRQRRKKRG-OH; Scrambled3, H-gvlavgrlivfhvlgvrvglfglrPPRRRQRRKKRG-OH.

The interfering peptides were manufactured by Shanghai Apeptide Co.,Ltd. at more than 99% purity and stored at -20 °C in powder aliquots of 1 mg until use to avoid freeze–thaw artefacts. Stock solutions of all peptides were prepared in sterile, ultrapure water supplemented with acetic acid at a final concentration of 1% (v/v). Briefly, lyophilized peptides were centrifuged, reconstituted to a concentration of 10 mM by gentle pipetting or low-speed agitation, and aliquoted for storage at -80°C. For the highly hydrophobic peptide EIP3, initial solubilization was performed in a minimal volume of DMSO to form a 50 mM concentrate prior to dilution in 1% acetic acid, ensuring the final DMSO concentration was negligible in cellular assays. For cell culture experiments, all peptide stock solutions were diluted into serum-free medium or PBS with vigorous mixing prior to their final dilution into complete cell culture media to prevent acid-induced precipitation. Vehicle controls containing an equivalent final concentration of acetic acid (and DMSO if applicable) were included in all experiments.

**Preparation of Ctrl-NPs or VAPG-NPs for EIP2 encapsulation**

The VSMC-targeting peptide (VAPG) with a C-terminal cysteine residue (VAPG-Cys) was synthesized by GL Biochem (Shanghai) with a purity >95%. The active targeting lipid conjugate (DSPE-PEG2000-VAPG) was synthesized via a thiol-maleimide click reaction. Briefly, DSPE-PEG2000-Mal and VAPG-Cys were dissolved in chloroform and dimethyl sulfoxide (DMSO), respectively. The solutions were combined at a molar ratio of 1:1.5 (DSPE-PEG2000-Mal:VAPG-Cys) in the presence of 0.1% (v/v) triethylamine and stirred under a nitrogen atmosphere for 24 hours at room temperature.

For VAPG-NPs, the lipid components—DSPC, cholesterol, DMG-PEG2000, DMG-PEG2000-VAPG, DMG-PEG2000-Cy5—were combined at a molar ratio of 50:40:6:3:1. For the Ctrl-NPs, DMG-PEG2000-VAPG was not used. In addition, DSPE-PEG2000-Cy5 was used to incorporate a fluorescent label. The organic solution was transferred to a round-bottom flask and evaporated to dryness under reduced pressure using a rotary evaporator to form a thin lipid film on the inner wall of the flask. The film was further desiccated under vacuum overnight to ensure complete removal of any residual organic solvent.

The dried lipid film was then hydrated with ultrapure water (for non-loaded NPs) or EIP2 solution (1.0 mg/mL in ultrapure water) by gentle agitation in a water bath above the phase transition temperature of the lipids. The resulting multilamellar vesicle suspension was sonicated in a water bath to detach the lipid film from the flask wall and then transferred to a tube. Subsequently, the suspension was subjected to probe sonication on ice to reduce the particle size and facilitate the formation of small unilamellar vesicles. The crude nanoparticle suspension was sequentially extruded through 0.45 μm and 0.22 μm aqueous syringe filters to achieve a uniform particle size distribution and ensure sterility. The purified nanoparticle suspension was stored at 4 °C for further use. The nanoparticle size and zeta potential are provided in Table S7, and the drug encapsulation efficiency of the drug-loading nanoparticles is summarized in Table S8.

**Flow cytometry analysis of NPs enrichment across vascular layers**

Sham operated and 5/6 nephrectomy mice were intravenously injected with EIP2-loaed Ctrl-NPs or EIP2-loaed VAPG-NPs, respectively. After circulation for 12h, animals were perfused with PBS to clear blood and unbound particles. The entire aorta was then excised and meticulously cleaned of perivascular adipose tissue. Preparation of Aortic Single-Cell Suspension. Isolated aortas were minced and enzymatically digested in a cocktail of Collagenase Type IV and Elastase at 37℃. The digested tissue was filtered through cell strainers (70 μm followed by 40 μm). Erythrocytes were lysed, and the resulting single-cell suspension was washed and resuspended in FACS buffer for analysis. Single-cell suspensions were Fc-blocked and then stained with a panel of fluorescently conjugated antibodies: anti-CD31-PE-Cy7 (#561410, BD Pharmingen™) for endothelial cells (tunica intima), anti-α-SMA-Alexa Fluor™ 488 (#53-9760-82, Invitrogen) for smooth muscle cells (tunica media) and anti-CD90.2-PE (#553014, BD Pharmingen™) for adventitial cells (tunica externa). Samples were analyzed by flow cytometry. The gating strategy sequentially identified live cells, singlets, and then the specific vascular layer populations. Cy5 fluorescence was measured within each population, with Median Fluorescence Intensity (MFI) serving as the primary metric for NPs enrichment.

**Statistical analysis**

Analyses were performed by using GraphPad Prism version 8.0.1. Data are expressed as mean±SEM. For animal study, all the data with n ≥ 6 were tested for normality using an Aderson-Darling, D’Agostino-Pearson, Kolmogorov-Smirnov or Shapiro-Wilk test. For in vitro experiments like western blot or qPCR, as each experimental data set is an average of a large number of cultured cells, we assumed the data were normally distributed based on the central limit theorem. For normally distributed data, differences between treatment groups were determined using unpaired t-test for two groups of data and one-way or two-way ANOVA for multiple groups of data. Values of P < 0.05 were considered statistically significant.
